# A framework based on subject-specific musculoskeletal models and Monte Carlo simulations to personalize muscle coordination retraining

**DOI:** 10.1038/s41598-024-53857-9

**Published:** 2024-02-12

**Authors:** Hans Kainz, Willi Koller, Elias Wallnöfer, Till R. Bader, Gabriel T. Mindler, Andreas Kranzl

**Affiliations:** 1 Department of Biomechanics, Kinesiology and Computer Science in Sport, Centre for Sport Science and University Sports, University of Vienna, Auf der Schmelz 6a (USZ II), 1150 Vienna, Austria; 2 Neuromechanics Research Group, Centre for Sport Science and University Sports, University of Vienna, Vienna, Austria; 3 Vienna Doctoral School of Pharmaceutical, Nutritional and Sport Sciences, University of Vienna, Vienna, Austria; 4 Department of Radiology, Orthopaedic Hospital Speising, Vienna, Austria; 5 Department of Paediatric Orthopaedics and Foot Surgery, Orthopaedic Hospital Speising, Vienna, Austria; 6 Vienna Bone and Growth Center, Vienna, Austria; 7 Laboratory for Gait and Movement Analysis, Orthopaedic Hospital Speising, Vienna, Austria

**Keywords:** Computational biology and bioinformatics, Biomedical engineering

## Abstract

Excessive loads at lower limb joints can lead to pain and degenerative diseases. Altering joint loads with muscle coordination retraining might help to treat or prevent clinical symptoms in a non-invasive way. Knowing how much muscle coordination retraining can reduce joint loads and which muscles have the biggest impact on joint loads is crucial for personalized gait retraining. We introduced a simulation framework to quantify the potential of muscle coordination retraining to reduce joint loads for an individuum. Furthermore, the proposed framework enables to pinpoint muscles, which alterations have the highest likelihood to reduce joint loads. Simulations were performed based on three-dimensional motion capture data of five healthy adolescents (femoral torsion 10°–29°, tibial torsion 19°–38°) and five patients with idiopathic torsional deformities at the femur and/or tibia (femoral torsion 18°–52°, tibial torsion 3°–50°). For each participant, a musculoskeletal model was modified to match the femoral and tibial geometry obtained from magnetic resonance images. Each participant’s model and the corresponding motion capture data were used as input for a Monte Carlo analysis to investigate how different muscle coordination strategies influence joint loads. OpenSim was used to run 10,000 simulations for each participant. Root-mean-square of muscle forces and peak joint contact forces were compared between simulations. Depending on the participant, altering muscle coordination led to a maximum reduction in hip, knee, patellofemoral and ankle joint loads between 5 and 18%, 4% and 45%, 16% and 36%, and 2% and 6%, respectively. In some but not all participants reducing joint loads at one joint increased joint loads at other joints. The required alteration in muscle forces to achieve a reduction in joint loads showed a large variability between participants. The potential of muscle coordination retraining to reduce joint loads depends on the person’s musculoskeletal geometry and gait pattern and therefore showed a large variability between participants, which highlights the usefulness and importance of the proposed framework to personalize gait retraining.

## Introduction

Altered loads at lower limb joints can lead to pain and degenerative joint diseases, e.g. osteoarthritis^1–3^. Measuring in-vivo joint loads is only possible with instrumented implants and therefore limited to a few studies with a small number of elderly participants^4–6^. Musculoskeletal modelling based on three-dimensional motion capture data enables the estimation of in-vivo joint loads during dynamic movements^7,8^.

Joint loads mainly depend on a person’s gait pattern, musculoskeletal geometry and muscle coordination. Numerous studies compared people with different gait pattern, e.g. typical versus pathological gait, and evaluated the impact on muscle forces and joint loads^9–12^. Several studies highlighted the impact of the subject-specific musculoskeletal geometry on hip and knee joint contact forces^13–17^. Modenese et al.^14^ showed that personalizing the femoral anteversion angle leads to more accurate estimations of knee joint contact forces. Furthermore, their study showed that joint loads increase with high anteversion angles, which is in agreement with our previous work^13,18^. Last but not least, joint loads depend on a person’s muscle coordination. The same walking pattern but different muscle recruitment strategies can influence muscle forces and joint loads as shown in many studies based on electromyography-informed musculoskeletal simulations^19–23^.

Gait retraining has the potential to alter joint loads in a non-invasive way^24–26^. Two different approaches have been previously proposed: (1) gait retraining with a focus on altering the gait pattern, i.e. joint kinematics^27^, and (2) gait retraining with a focus on altering muscle coordination^28^. While much research efforts focused on the first approach^24–27,29–31^, muscle coordination retraining has only been studied in a few papers^28,32,33^. DeMers et al.^33^ and Uhlrich et al.^28^ showed that altering lower limb muscle activations can reduce knee joint loads. These studies, however, were based on generic-scaled models and therefore did not account for the individuum’s’ musculoskeletal geometry. Van Veen et al.^32^ estimated joint loads in four participants using two objective functions. The first one minimized overall muscle activation, whereas the second one minimized the magnitude of the joint contact forces. Their results showed that alternative muscle coordination can reduce the loading at the hip and knee but not at the ankle joint. Furthermore, their study showed that joint loads at non-targeted joints increased when aiming to minimize the load at an adjacent joint^32^. All these studies related to muscle coordinate retraining^28,32,33^ only focused on a limited number of possible muscle coordination strategies to minimize joint loads. The redundancy of our musculoskeletal system, however, enables to generate the same movement with an infinite number of different muscle coordination strategies. Hence, the whole potential of muscle coordination retraining to reduce joint loads has not been evaluated yet.

In this paper we introduce a framework based on medical-images informed musculoskeletal models and Monte Carlo simulations which enables to (i) quantify the potential of muscle coordination retraining to reduce joint loads for a specific person and (ii) pinpoint muscles which alterations have the highest likelihood to reduce joint loads. Using this framework, we aimed to enhance our insights in muscle coordination retraining by addressing the following research questions: (1) How much can muscle coordination retraining reduce hip, knee, patellofemoral and ankle joint loads? (2) How does reducing loads at one joint affect loads at the non-target joints? (3) Which muscles should be targeted during gait retraining to reduce joint loads? Considering that the musculoskeletal geometry and gait pattern differs between people, we hypothesized that the potential to reduce joint loads will vary between people. Furthermore, we assumed that muscle recruitment strategies to obtain a reduction in joint loads will vary between people.

## Methods

**Figure 1:**
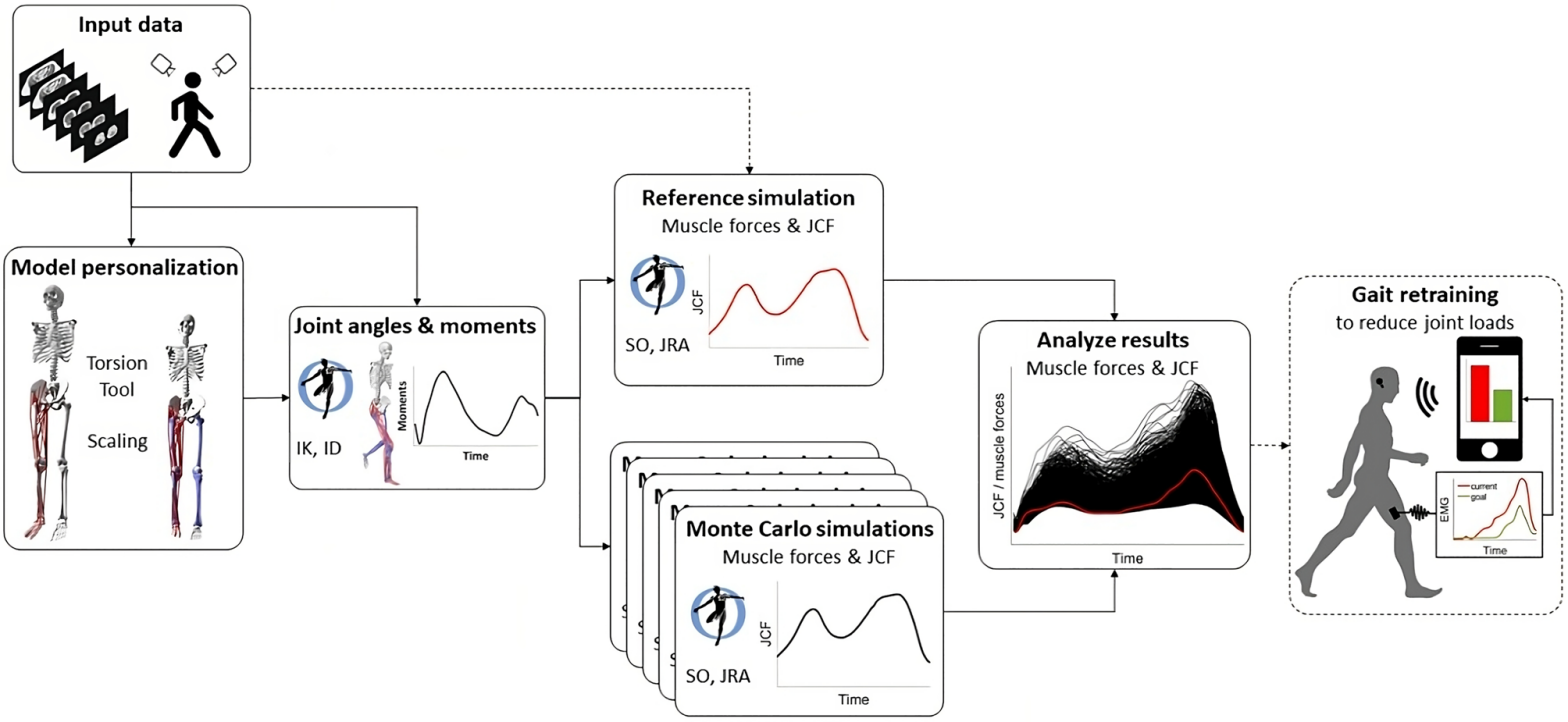
Schematic illustration of the framework. Medical images and three-dimensional motion capture data were used for subject-specific personalization of the musculoskeletal model. Joint angles and moments were calculated in OpenSim using inverse kinematics (IK) and inverse dynamics (ID). Static optimization (SO) with an equal weighting of all muscles and joint reaction analyses (JRA) were used to estimate muscle forces and joint contact forces (JCF) for the reference simulation. Monte Carlo simulations (10,000 simulations) were performed to evaluate how different muscle coordination strategies influence muscle and JCF. Results from the Monte Carlo simulations were compared to the reference simulation to investigate which muscles should be targeted during the gait retraining to reduce joint loads. In the future, we plan to use electromyography-informed simulations for the reference simulation (indicated with the dashed line between input data and reference simulation). Furthermore, we plan to use this workflow to inform subject-specific gait retraining for people with various pathologies with the aim to reduce joint loads and potentially decrease pain or delay the onset of osteoarthritis (indicated with the dashed box).

We developed a framework based on personalized medical imaging-informed musculoskeletal models and Monte Carlo simulations to address our research questions (Fig. 1). Simulations were performed based on subject-specific models and three-dimensional motion capture data of ten participants. For each participant 10,000 simulations were performed to assess how different muscle coordination strategies influence joint loads.

### Participants

**Figure 2:**
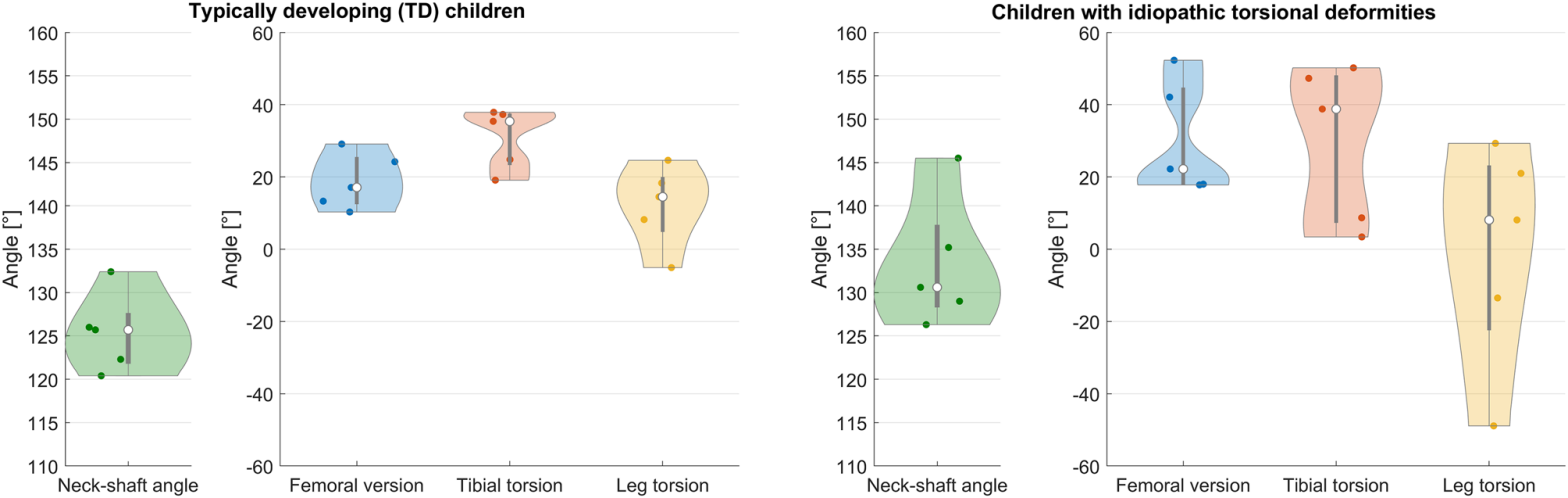
Neck-shaft angle (NSA), femoral version (FV), tibia torsion (TT) and whole leg torsion (LT = TT-FV) from the right leg of our typically developing children (left subplots) and participants with idiopathic torsional deformities (right subplots). NSA, FV and TT were quantified from magnetic resonance images using commonly used three-dimensional measurement techniques^37,38^. Simulations for the right leg were performed for each participant. Geometrical information for the left leg is provided in the electronic appendix.

Previous collected magnetic resonance images (MRI) and three-dimensional motion capture data of five typically developing (TD) children and five children with idiopathic torsional deformities (Table 1, Fig. 2) were used to create subject-specific models and run musculoskeletal simulations. These two participant groups were selected because it enabled us to evaluate the influence on our findings due to (i) different musculoskeletal geometries but similar TD gait pattern and (ii) different musculoskeletal geometries and pathological gait patterns, i.e. in-toeing and out-toeing gait. Furthermore, the necessary data, i.e. medical images and three-dimensional motion capture data were available from these participants. Written informed consent was obtained from the legal guardians or parents of our participants for using their data, i.e. MRI images and motion capture data, for research purposes. All methods were carried out in accordance with relevant guidelines and regulations.

**Table 1:** Characteristics of the participants with idiopathic torsional deformities (PA01–PA05) and typically developing participants (TD01–TD05).

ID	Weight [kg]	Height [mm]	Age [years]	Gender	Walking velocity [m/s]
PA01	32.9	1220	7	Female	1.37
PA02	39.3	1460	9	Male	1.26
PA03	55.9	1585	14	Male	1.36
PA04	40.9	1485	9	Male	1.5
PA05	66.3	1560	11	Male	1.03
TD01	38.9	1470	10	Female	1.48
TD02	53	1630	13	Male	1.12
TD03	54.8	1615	15	Female	1.06
TD04	59.3	1620	18	Female	0.93
TD05	29.6	1370	11	Male	1.47

### Experimental data

Details for the data collection from magnetic resonance images (MRI) and three-dimensional (3D) motion capture data of the five TD children are available in previous publications^34,35^. In short, MRIs of the pelvis and lower limbs were collected using a 1.5 Tesla MRI scanner (MAGNETOM Avanto, Siemens, Berlin/Munic, Germany) with a slice thickness of 1.1 mm and slice spacing of 1.1 mm. Motion capture data were collected using an 8-camera 3D motion capture system (Vicon Motion Systems, Oxford, UK) with an extended Plug-in Gait marker set^36^. Ethics approval was obtained from the Queensland Children’s Health Services Human Research Ethics Committee (HREC/13/QRCH/197).

MRI and 3D motion capture data from the participants with idiopathic torsional deformities were previously collected at the Orthopedic Hospital Speising, Vienna, Austria. In our clinical center people are classified as patient with idiopathic torsional deformities based on a comprehensive assessment which includes medical images, clinical examination and three-dimensional gait analysis. Gait analysis data were collected using an 17-camera motion capture system (Vicon Motion Systems, Oxford, UK) with a modified Cleveland model marker set^39^. MRI examination were performed on a 1.5 T scanner (Magnetom Sola, Siemens, Erlangen, Germany) in supine position. Axial images were acquired in one imaging session covering both hip joints, both knee joints, and both ankle joints using a 16-channel body coil for both hips and knees, and a head-and-neck coil for both ankles. The legs were fixated with elastic straps to prevent motion during scanning. MR images were acquired in stacks of T2-weigthed TSE sequences (TR 4000 ms, TE 69 ms, slice thickness 5 mm, gap 10%, FOV 380 mm, voxel size 0.6 × 0.6 × 5 mm). The local ethics committee approved the use of the retrospective data (EK022020).

### Musculoskeletal simulations

The modified generic Rajagopal model^28,40^ was used as the musculoskeletal base model for all simulations. This model included 21 degrees of freedom and 40 musculotendon actuators for each leg. For each participant, the femoral neck-shaft angle (NSA), femoral version (FV) and tibia torsion (TT) were quantified from the MRI images using commonly used three-dimensional measurement techniques^37,38^. In short, anatomical landmarks at the femur were selected to define the femoral neck axis, shaft axis and knee axis, which were used to calculate the NSA and FV. Anatomical landmarks at the tibia and fibula were used to define a tangential axis at the back of the tibia and transmalleolar axis, which were used to calculate TT. The same techniques were used to quantify the NSA, FV and TT of the Rajagopal model. We modified the femoral and tibial geometry of the base model to match each participant’s NSA, FV and TT using the recently developed Torsion Tool^41^. The original Torsion Tool only worked for the gait2392 model. Hence, we updated the Torsion Tool, which should now work with the majority of available OpenSim models. The updated script is freely available on https://simtk.org/projects/torsiontool. Notably, the personalization of the bony geometry also altered the attachment points of muscles and consequently lines of action and moment arms of several muscles. After personalizing the geometry, each model’s segment dimensions and muscle–tendon properties (i.e. optimal fiber length and tendon slack length) were linearly scaled to the anthropometry of each participant. Scale factors for femur lengths, tibia lengths and pelvic width (distance between hip joint centers) were derived from the magnetic resonance images. The remaining scale factors, i.e. feet, torso and pelvic depth, were derived from the surface marker locations.

Monte Carlo analyses were used to investigate the impact of different muscle coordination strategies on muscle forces and joint contact forces (JCF). First, each participant’s model and the corresponding motion capture data were used to calculate joint angles and joint moments using OpenSim’s Inverse Kinematics and Inverse Dynamic Tools^42^. Second, we plotted the moment arms and muscle length from all models to verify that muscle–tendon kinematics is reasonable, i.e. does not lead to discontinuities during the walking pattern of our participants. For all participants, dynamic muscle moment arms and muscle–tendon lengths showed smooth waveform throughout the whole gait cycle. Finally, Monte Carlo analyses were performed based on the personalized musculoskeletal models and obtained joint kinematics and joint kinetics. A modified static optimization approach^28^, which allowed to allocate different penalty weights to each muscle (${w}_{i}$) while minimizing the sum of squared muscle activation (${a}_{i}^{2}$, Eq. 1), was used to calculate muscle forces and JCF. Penalty weights of 1 (= no penalty), 10 and 100 were randomly selected for all muscles of the right leg. The same random combination of muscle weights (10,000 different combinations) was used for each participant. To decrease the computational time, we calculated muscle forces only for the right leg. The left leg and torso were actuated with nine ideal torque actuators. Parallel computing in MATLAB (R2023a, MathWorks, Natick, MA, United States) was used to run the 10,000 simulations for each participant. For the reference simulation, muscle forces and JCF were calculated using static optimization with an equal weighting (${w}_{i}=1$) for all muscles, which is a commonly used approach to estimate muscle forces in people without neurological disorders^28,43,44^. Notable, JCF in OpenSim are calculated in a post-processing procedure based on the obtained joint kinematics and muscle forces^45^, which is a widely used approach^10,28,44,46^ and has been shown to provide physiological plausible results with an acceptable accuracy^14^.

1 $$min\left(\sum_{i=1}^{nMuscles}{w}_{i}{a}_{i}^{2}\right)$$

### Data analyses

Root-mean-square of muscle forces and peak JCF were compared between simulations. To quantify the possible reduction in joint loads due to muscle coordination retraining, we compared simulations which resulted in the lowest JCF with the reference simulations. Furthermore, to evaluate how reducing JCF at one joint (e.g. hip), alters JCF at the other joints (e.g. knee, patellofemoral and ankle), we compared JCF from all joints between simulations with the lowest JCF at a specific joint (e.g. hip) and the reference simulations. To evaluate which muscles should be targeted during gait retraining to reduce JCF, we compared root-mean-square (RMS) of muscles forces between simulations with the lowest JCF to the values from the reference simulations. To assess the sensitivity of our results we additionally conducted all analyses for the simulations which resulted in the 50 lowest JCF. Only descriptive statistics was used for the presentation of our findings due to the explorative nature of our research design.

## Results

**Figure 3:**
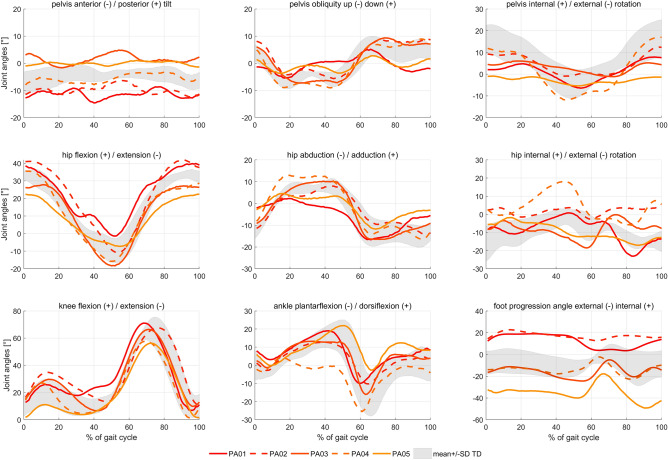
Joint kinematics of the participants with idiopathic torsion deformities. Grey area represents the mean ± one standard deviation values from our typically developing (TD) participants.

Joint kinematics of our TD participants were in agreement with previously reported values^47^. Two participants with idiopathic torsional deformities, i.e. PA01 and PA02, walked with an in-toeing gait pattern whereas all other patients walked with externally rotated feet (Fig. 3). Inverse kinematic tracking errors were within the OpenSim recommendations^48^. Computational time for the Monte Carlo simulations were approximately 23 h for the 10,000 simulations for each participant on a standard working computer (2.4 GHz, 8 cores, 32 GB RAM). The mean values of the predicted decrease in JCF were less than 0.5% different when calculated using 9000 versus 10,000 simulations, indicating that an adequate number of simulations were performed for the purpose of our study^49^. Differences in analyses based on 9000 versus 10,000 simulations would not have changed the findings and conclusions from our study (see supplementary figures).

**Figure 4:**
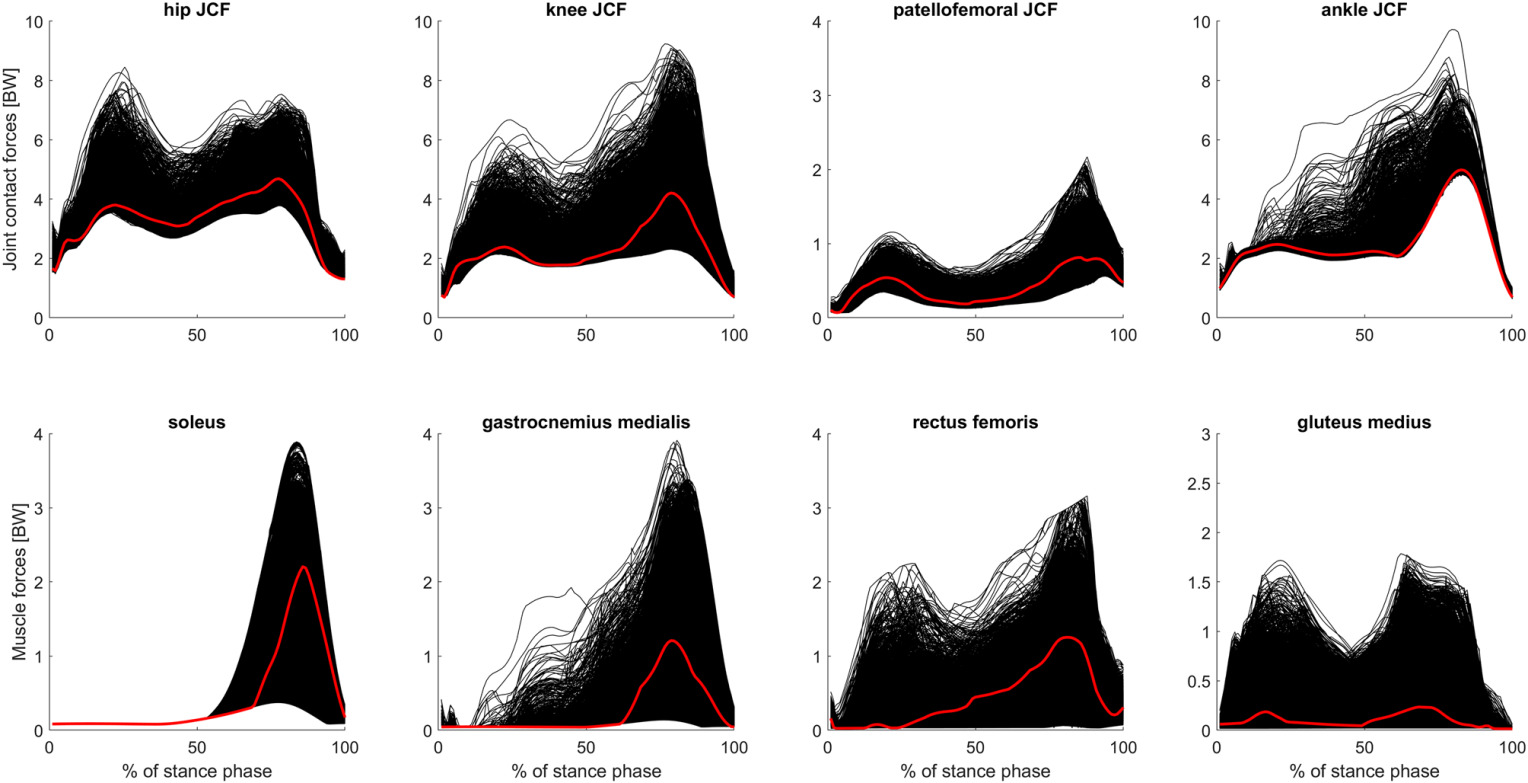
Joint contact forces (JCF) and an example of muscle forces obtained from one participant (PA05). Black waveforms are the results from the 10,000 Monte Carlo simulations. Red waveforms are the results from the reference simulation. Example plots of a healthy participant are provided in the electronic appendix.

**Figure 5:**
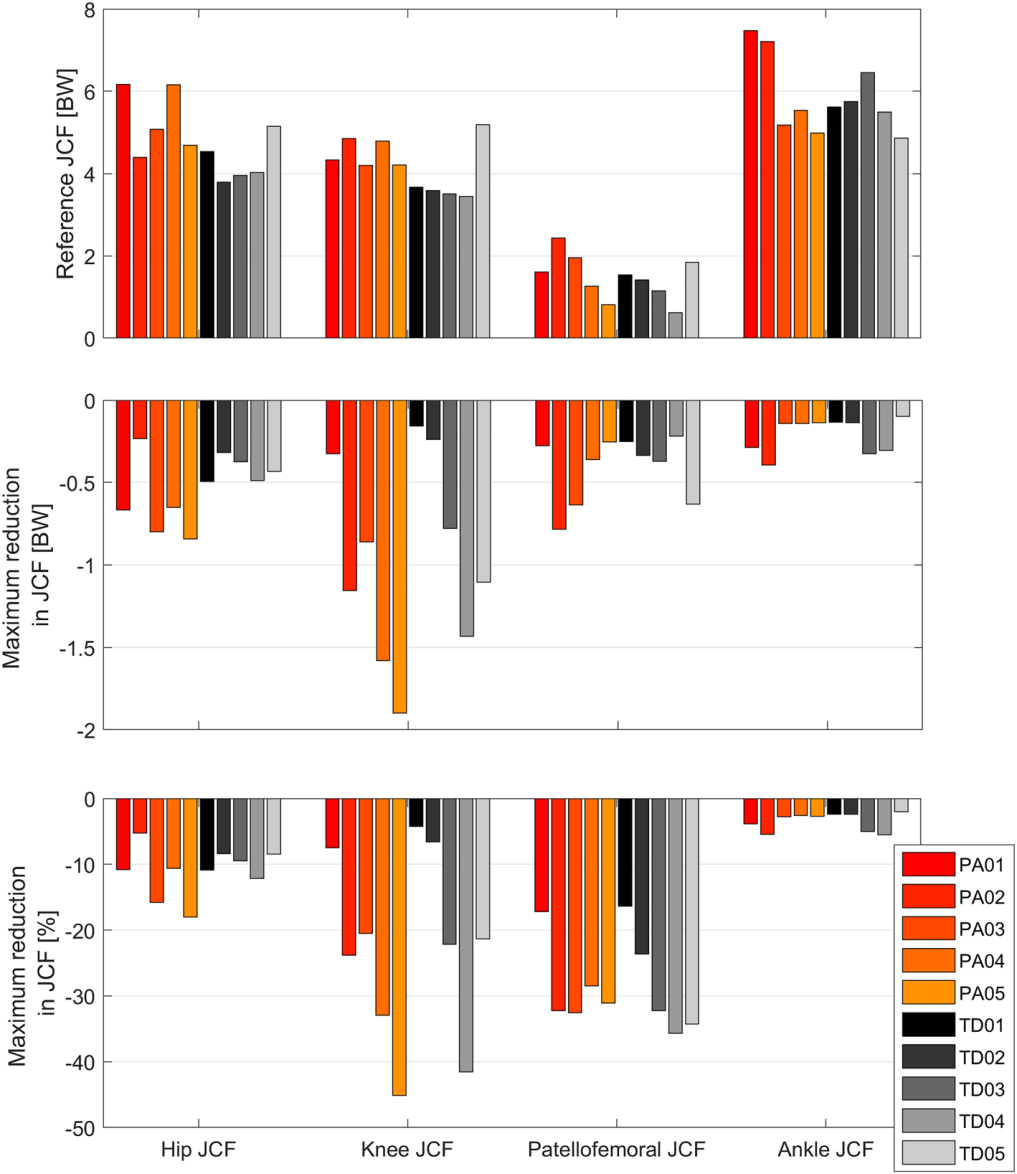
Joint contact forces (JCF) from the reference simulations based on static optimization with an equal weighting of all muscles (top subplot) and maximum reduction in JCF obtained from the Monte Carlo simulations. BW: body weight.

**Figure 6:**
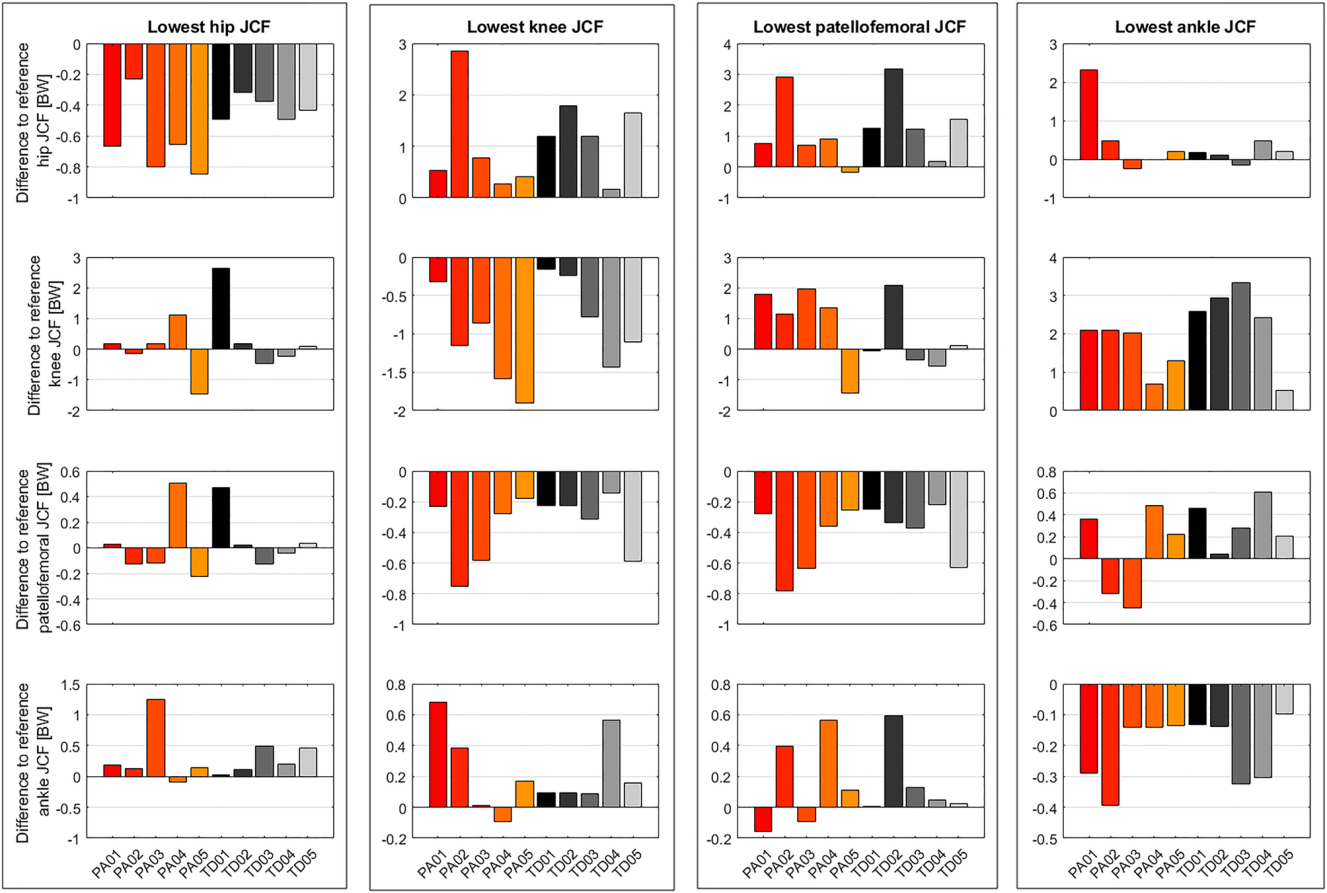
Difference to reference joint contact forces (JCF) for simulation with the lowest hip (first column), knee (second column), patellofemoral (third column) and ankle (fourth column) JCF and the influence on JCF at other joints. Each column of subplots is based on the same simulations. BW: body weight.

**Figure 7:**
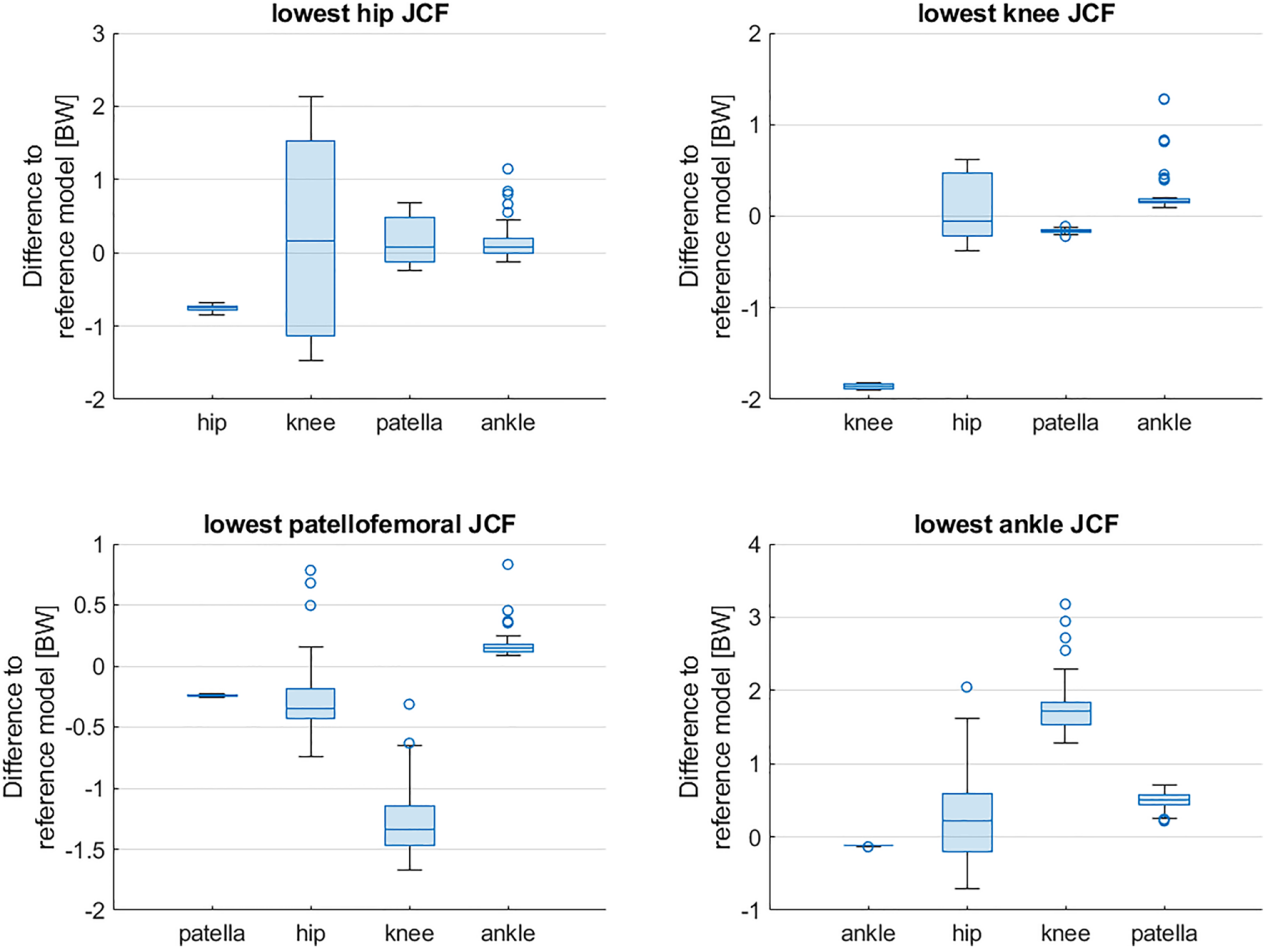
Difference to reference joint contact forces (JCF) for simulations with the 50 lowest hip, knee, patellofemoral and ankle JCF and the influence on JCF at other joints. Data from one participant (PA05) are shown in this figure. Plots from all other participants are provided in the supplementary material. BW: body weight.

Reference simulations led to peak (mean ± standard deviation over all participants) hip, knee, patellofemoral and ankle JCF of 4.8 ± 0.8 body weight (BW), 4.2 ± 0.6 BW, 1.5 ± 0.5 BW and 5.9 ± 0.9 BW, respectively. On average the Monte Carlo simulations reduced hip, knee, patellofemoral and ankle JCF in 26 ± 12%, 27 ± 13%, 22 ± 14% and 26 ± 9% out of the 10.000 simulations, respectively. Depending on the participant, altering muscle coordination led to a maximum reduction in hip, knee, patellofemoral and ankle JCF between 5 and 18%, 4% and 45%, 16% and 36%, and 2% and 6%, respectively (Figs. 4 and 5). Simulations with the lowest hip JCF in our study, increased knee and patellofemoral JCF in two participants (PA04 and TD01), whereas in all other participants knee and patellofemoral JCF either decreased or remained similar (increased less than 5%) to the reference simulation (Fig. 6). Simulations with the lowest knee and patellofemoral JCF in our study, increased hip and ankle JCF in most participants but with different magnitudes. Simulations with the lowest ankle JCF in our study, increased knee and patellofemoral JCF in most participants and hip JCF in one participant. Analyzing simulations with the 50 lowest JCF in our study showed a similar reduction in JCF but the influence on other joints showed a larger variability (Fig. 7).

**Figure 8:**
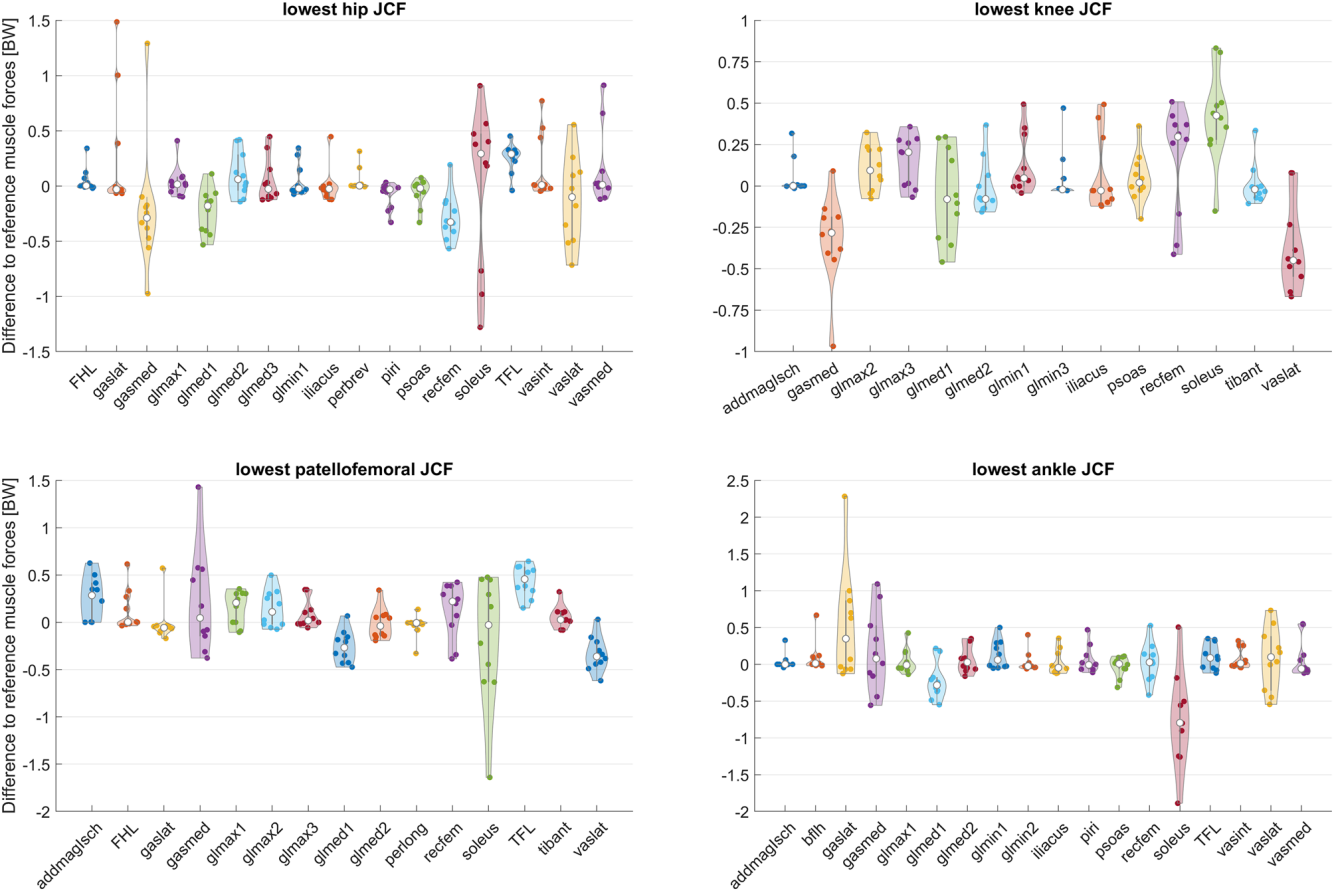
Difference to reference root-mean-square (RMS) muscle forces for simulations with the lowest joint contact forces (JCF). A selection of muscles with a difference of at least 0.3 body weight [BW] in at least one participant are included in this figure. Figures with all muscles are provided in the electronic appendix of this paper. Each dot in the violin plot represents the result from one participant. FHL: flexor hallucis longus; TFL: tensor fasciae latae.

**Figure 9:**
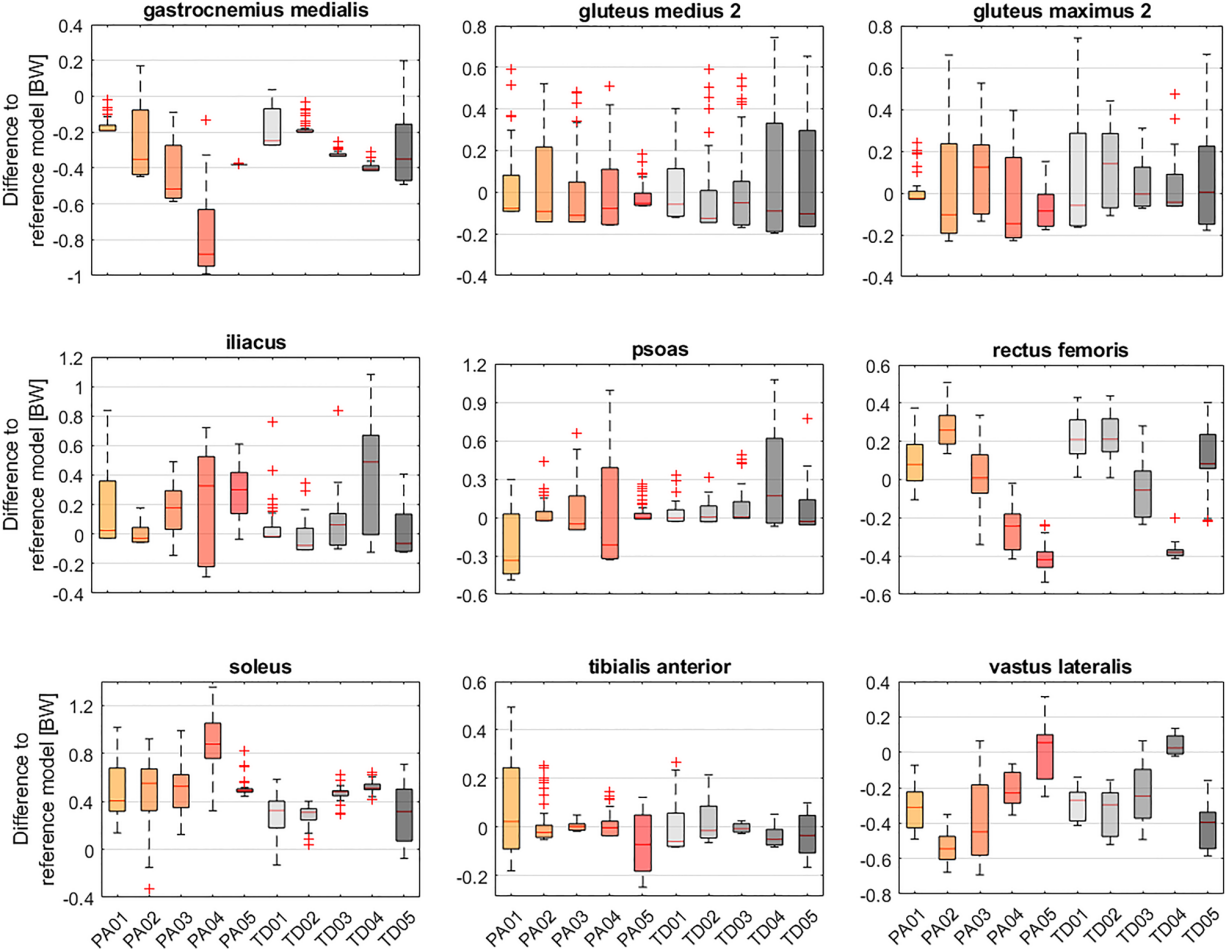
Difference to reference root-mean-square (RMS) muscle forces for simulations with the 50 lowest knee joint contact forces (JCF). A selection of muscles is shown in this figure. Similar figures for simulations with the lowest hip, patellofemoral and ankle JCF are provided in the electronic appendix. BW: body weight.

Depending on the muscle and joint, a varying degree of variability in the adjustment of muscle forces to achieve a reduction in JCF was observed among participants (Fig. 8). In most participants, rectus femoris and gastrocnemius medialis forces were reduced and tensor fasciae latae forces were increased in simulations with the lowest hip JCF (Fig. 8). In simulations with the lowest knee JCF, gastrocnemius medialis and vastus lateralis forces were reduced and gluteus maximus and soleus forces were increased for most participants. In simulations with the lowest patellofemoral JCF, vastus lateralis forces were reduced and tensor fasciae latae and adductor magnus forces were increased for most participants. Increased gastrocnemius lateralis and decreased soleus forces were observed in most participants for simulations with the lowest ankle JCF. Analyzing simulations with the 50 lowest JCF showed a low (e.g. rectus femoris from TD04) to larger (e.g. rectus femoris from PA03) sensitivity of the alteration in muscles forces needed to reduce JCF (Fig. 9).

## Discussion

We introduced a framework based on personalized musculoskeletal models and Monte Carlo simulations which enabled us to (i) quantify the potential of muscle coordination retraining to reduce joint loads for a specific person and (ii) pinpoint muscles which alterations have the highest likelihood to reduce joint loads. Using this framework, we showed that the potential to reduce joint loads with altered muscle coordination varies a lot between people (e.g. knee JCF between 5 and 45%). Furthermore, we showed that a decrease in joint loads requires a subject-specific adaptation of muscle coordination. Both of these findings were in agreement with our hypotheses and highlight the usefulness and importance of the proposed workflow to personalize gait retraining.

Previous studies investigated the impact of altered muscle coordination on knee JCF. DeMers et al.^33^ evaluated how different muscle coordination strategies influence knee JCF based on data of one person with an instrumented total knee replacement. They showed that knee JCF could be reduced by increasing gluteus medius, psoas, iliacus and soleus forces, and by decreasing gastrocnemius and rectus femoris forces. In our study a reduction in knee JCF was achieved by increasing gluteus medius, psoas, iliacus and soleus in 40%, 50%, 30% and 90% of participants, respectively and decreasing gastrocnemius and rectus femoris forces in 90% and 30% of participants, respectively. Hence, the findings of some but not all our participants agree with the previous investigation based on one elderly person^33^. Uhlrich et al.^28^ recently showed that reducing the ratio of gastrocnemius-to-soleus muscle activity can decrease knee JCF. In our study reducing gastrocnemius and increasing soleus forces decreased knee JCF in all except of one participant and therefore confirms the previously obtained findings^28^.

Our findings largely agree with previous investigation based on generic-scaled models^28,33^, which did not take the subject-specific musculoskeletal geometry into account. Some muscle (e.g. soleus and gastrocnemicus for reducing knee JCF) seem to be less affected by the subject-specific geometry and showed a consistent pattern over most participants. Other muscles (e.g. gluteus medius and psoas for reducing knee JCF), however, showed heterogeneous results. For example, gluteus medius forces had to be decreased in six and increased in four of our participants to reduce knee JCF, which highlights the varied coordination strategies between people to reduce JCF (Fig. 8). Interestingly, although increasing soleus forces decreased knee JCF in most participants (9 out of 10), it only decreased patellofemoral JCF in 50% of our participants. This finding indicates that muscle coordination strategies that minimize knee JCF do not necessarily decrease patellofemoral JCF.

Altered muscle coordination had a big impact on knee and patellofemoral JCF, a small impact on hip JCF and a negligible impact on ankle JCF. In our participants a maximum reduction of hip, knee, patellofemoral and ankle JCF of 18%, 45%, 36% and 6% was observed, respectively (Fig. 5). These findings are in agreement with Van Veen et al.^32^, who showed that altered muscle coordination can lead to a large, medium and negligible reduction in JCF at knee, hip and ankle joint, respectively. Hence, it seems that muscle coordination retraining has the potential to reduce hip, knee and patellofemoral JCF but not ankle JCF.

Decreasing JCF at one joint did not necessarily increase JCF at other joints. In our simulations, decreasing hip JCF had a neglectable impact on JCF at other joints for most participants (except PA04 and TD01). Decreasing knee or patellofemoral JCF, however, increased hip JCF in most participants but with varied magnitudes (Fig. 6). Comparable results were observed by Van Veen et al.^32^, who investigated the impact of altered muscle coordination on JCF in four participants. Similar to our study, the simulations from Van Veen et al.^32^ were mostly (3 out of 4 participants) based on medical imaging informed musculoskeletal models. In their study, a reduction of JCF was obtained with different muscle coordination strategies between participants, which confirmed the findings of our study. For example, in some participants a reduction in hip JCF was achieved with an active gluteus medius muscle whereas in other participants the gluteus medius was not active in simulations with reduced hip JCF^32^. Their and our findings highlight the large variability between participants and the importance of including medical imaging informed musculoskeletal models for tailoring gait retraining to a specific person.

A recent study used Monte Carlo simulations to investigate the sensitivity of patellofemoral JCF to uncertainties in muscle recruitment strategies^15^. Their study showed that increased rectus femoris forces increase patellofemoral JCF, which is in agreement with the findings in most but not all of our participants. Simulations from Wheatley et al.^15^ were based on a limited experimental data set and, therefore, did not include subject-specific musculoskeletal models and gait analysis data, which might explain the partly contrary results between their study and our results. A further study based on motion capture data of a single participant and Monte Carlo simulations showed that altering muscle coordination is insufficient to restore normative knee loading in knees with a deficient anterior crucial ligament or menisci^50^, which indicates the importance of healthy anatomical structures.

Our study involved participants walking at velocities ranging from 0.9 m/s to 1.5 m/s. Prior research has indicated that even slight variations in walking velocity within this range can lead to an approximate alteration of hip and knee JCF by about one body weight^51,52^. Notably, despite the variation in walking velocities, JCF from our reference simulations were very similar among most participants (e.g., TD01, TD02, TD03, and TD04), underscoring the substantial influence of bony geometry on JCF^53^. While we observed large differences in the maximum reduction of JCF, even among participants with similar walking velocities (e.g., TD01 vs. TD05), we assume that walking velocity had a relatively minor impact on our analyses. Nevertheless, further investigations are needed to validate this assumption.

In the future, altering joint loads with personalized muscle coordination retraining might help to treat or prevent clinical symptoms in a non-invasive way. For example, the direction of the hip JCF has been shown to determine typical and pathological growth plate stresses and bone growth^9,54^. Normalizing the direction of the hip JCF with personalized muscle coordination retraining at an early stage might, therefore, help to prevent the development of torsional femoral deformities. In people with mild hip or knee joint osteoarthritis personalized muscle coordination retraining might help to slow down the progression of osteoarthritis and therefore delay the need for a joint replacement. Hence, the proposed framework has the potential to improve clinical symptoms and therefore quality of life of thousands of people. However, more research efforts are needed before the proposed framework can be integrated in clinical routines.

We personalized a generic musculoskeletal model by incorporating anatomical details derived from MRI images. As a result, our models accurately considered key anatomical features (e.g., femoral neck-shaft angle), known to significantly influence simulation outcomes^53^, while overlooking others (e.g., femoral shaft bending, variations in muscle attachment points among participants). While fully MRI-based models^17,55,56^ theoretically offer a more precise representation of a participant's musculoskeletal geometry, they require defining muscle attachment points based on MRI images—a process fraught with challenges and uncertainties. Consequently, we opted against using this approach.

We did not minimize JCF in the cost function of our simulations; therefore, we do not know if our simulation results included solutions with the minimal possible JCF. Van Veen et al.^32^ estimated joint loads in four participants using objective functions that minimized the magnitude of the joint contact forces. In their study, and in agreement with our findings, increased tensor faciae latae activity led to the lowest hip JCF, decreased gastrocnemius and increased soleus activities led to lowest knee JCF, and increased gastrocnemius and decreased soleus activities led to the lowest ankle JCF. We observed the lowest hip JCF in simulations with decreased rectus femoris activity in most of our participants, whereas this was only the case in one out of four participant in the study from Van Veen et al.^32^. The partly different findings might be attributed to variation in study populations, i.e. participants with different musculoskeletal geometries and pathologies, as well as differences in the objective functions used for the simulations.

Our proposed workflow can be further improved by addressing the following limitations. First, we had no electromyography data and therefore we used static optimization to estimate muscle forces and subsequently JCF in our reference simulations. Minimizing squared muscle activations during static optimization is a surrogate for minimizing metabolic cost, has been widely used for gait simulations^44,57,58^ and showed reasonable agreement with experimentally measured electromyography data^45,59^ and JCF from instrumented joint replacement^60,61^. Hence, we believe the muscle forces and JCF from our reference simulations are reasonable. Nevertheless, electromyography informed simulation^19^ would account for the subject-specific motor control and, therefore, potentially increase the accuracy of our reference simulations^62^. Hence, for prospective studies we recommend to collect electromyography data and use electromyography-informed simulations to estimate the reference muscle forces and JCF. Second, we only calculated total knee JCF and did not divide the knee joint loads into the medial and lateral compartment. For many clinical applications, e.g. delay the onset or progression of knee osteoarthritis^63^, it might be important to separately assess medial and lateral compartment loads. In our proof-of-concept study we did not have a specific clinical research question and, therefore, we only estimated the total knee JCF. Depending on the research question, our workflow could be used with different musculoskeletal base models, e.g. Lerner model^64^, which allow estimating medial and lateral knee JCF. Third, evaluating if people are able to modify their muscle coordination as recommended in our simulations was beyond the scope of our study. In a recent study by Uhlrich et al.^28^, healthy individuals were able to change the ratio between gastrocnemius-to-soleus muscle activity by 25 ± 15% after a single session of muscle coordination retraining. Although these findings are very promising, future research is needed to evaluate which muscles can be easily altered with gait retraining and which training modalities achieve the best results. Fourth, JCF were calculated based on rigid-body simulations in OpenSim, a common approach known to yield reasonable results^14,53,65^, but one that neglects soft tissue contact biomechanics. Modeling approaches that combine musculoskeletal dynamics and joint-level finite element analysis in a concurrent framework can provide insights into the interaction between muscle activation and joint tissue deformation^66–68^. Such approaches might lead to more realistic JCF but were beyond the scope of the current study. Furthermore, muscle–tendon parameters, i.e. optimal fiber length and tendon slack length, were linearly scaled in our models but could be further personalized with more sophisticated methods^69,70^. Sixth, we arbitrary chose a n = 50 simulations to investigate the sensitivity of our simulations.

In summary, our simulation framework enables to quantify the potential of muscle coordination retraining to reduce joint loads for an individuum prior to the start of the intervention. Subject-specific models, which account for each participant’s musculoskeletal geometry, and Monte Carlo simulations, which allowed to evaluate the impact of a large number of possible muscle coordination strategies on muscle forces and JCF, were key elements in our framework. The magnitude of reduction in joint loads as well as the required alteration in muscle forces to achieve a reduction in joint loads showed a large variability between participants, which highlights the usefulness and importance of the proposed workflow to personalize gait retraining.

### Supplementary Information

Supplementary Figures.

## Data Availability

The anonymised experimental input data, i.e. motion capture data, for all simulations as well as the personalized musculoskeletal OpenSim models are available on https://github.com/HansUniVie/MuscleCoordinationRetraining. Magnetic resonance images, which were used to personalize the musculoskeletal model, are confidential but may be obtained with data use agreements. Researchers interested in access to the anonymised magnetic resonance images may contact Hans Kainz at hans.kainz@univie.ac.at. It may take several months to negotiate data use agreements and gain access to the data. The authors will assist with any reasonable replication attempts for 2 years following publication.

## Code availability

The updated Torsion Tool code for modifying the bony geometry in OpenSim models is available at https://simtk.org/projects/torsiontool. The used code for the Monte Carlo simulations and summarizing the simulations results is available at https://github.com/HansUniVie/MuscleCoordinationRetraining.
