# Supplementary material for: A framework based on subject-specific musculoskeletal models and Monte Carlo simulations to personalize muscle coordination retraining
Source: Sci Rep. 2024 Feb 12;14:3567. doi: 10.1038/s41598-024-53857-9 (PMC10861532; doi:10.1038/s41598-024-53857-9)
Supplement: Supplementary file 1 — Supplementary Figures. [file 41598_2024_53857_MOESM1_ESM.docx]

**Supplementary material**

A framework based on subject-specific musculoskeletal models and Monte Carlo simulations to personalize muscle coordination retraining

Hans Kainz^1,2,*^, Willi Koller^1,2,3^, Elias Wallnöfer^1,2,3^, Till R Bader^4^, Gabriel T Mindler^5,6^, Andreas Kranzl^6,7^

* Corresponding author:

Ass.-Prof. Mag. Hans Kainz, MSc PhD

[hans.kainz@univie.ac.at](mailto:hans.kainz@univie.ac.at)

Head of the Neuromechanics Research Group, University of Vienna

<https://neuromechanics.univie.ac.at/>

University of Vienna, Department of Biomechanics, Kinesiology and Computer Science in Sport, Centre for Sport Science and University Sports

Auf der Schmelz 6a (USZ II), 1150 Vienna, Austria

^1^ Department of Biomechanics, Kinesiology and Computer Science in Sport, Centre for Sport Science and University Sports, University of Vienna, Vienna, Austria

^2^ Neuromechanics Research Group, Centre for Sport Science and University Sports, University of Vienna, Vienna, Austria

^3^ Vienna Doctoral School of Pharmaceutical, Nutritional and Sport Sciences, University of Vienna, Vienna, Austria

^4^ Department of Radiology, Orthopaedic Hospital Speising, Vienna, Austria

^5^ Department of Paediatric Orthopaedics, Orthopaedic Hospital Speising, Vienna, Austria

^6^ Vienna Bone and Growth Center, Vienna, Austria

^7^ Laboratory for Gait and Movement Analysis, Orthopaedic Hospital Speising, Vienna, Austria


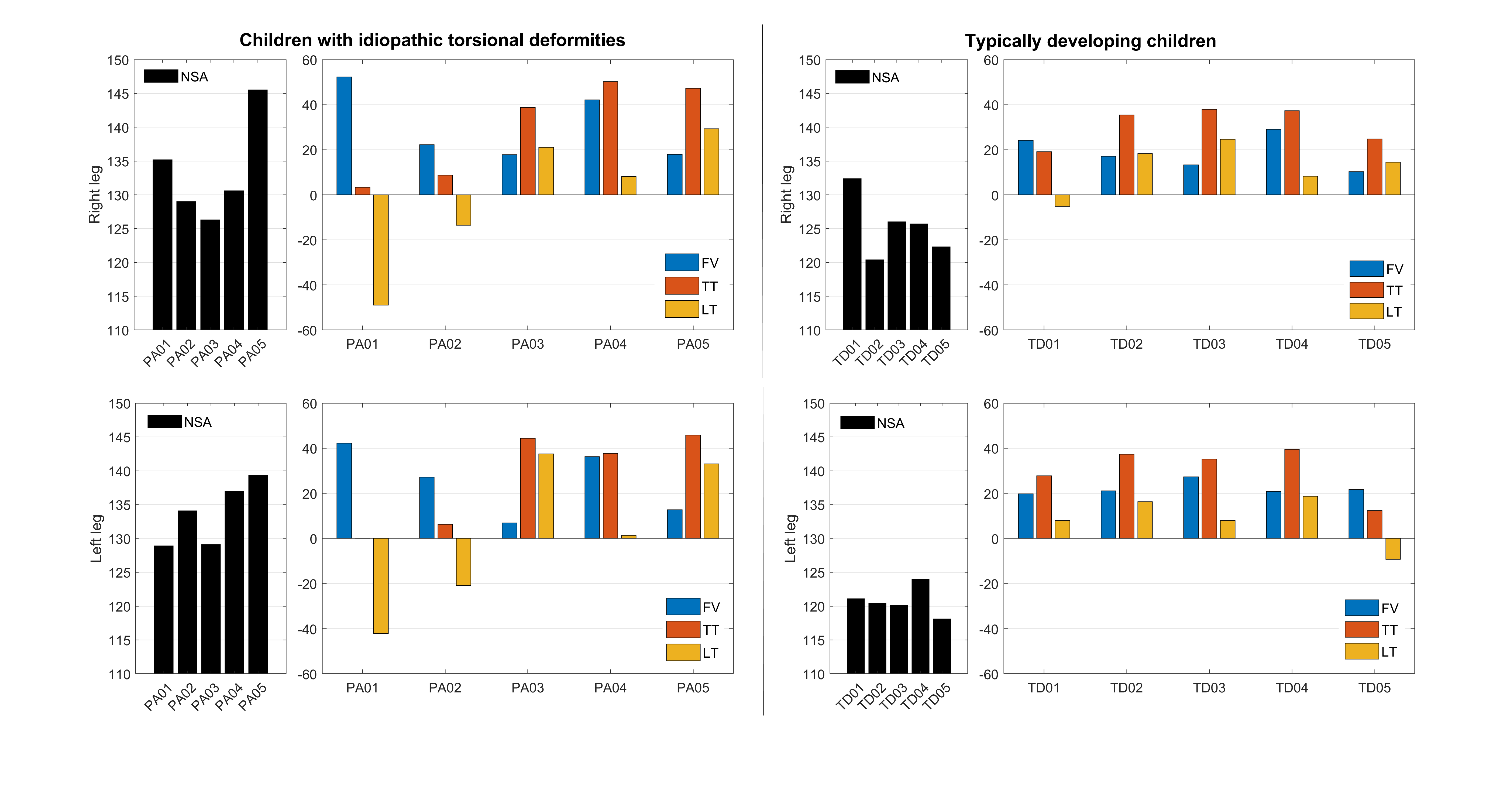


**Figure S1.** Neck-shaft angle (NSA), femoral version (FV), tibia torsion (TT) and whole leg torsion (LT=TT-FV) from our participants with idiopathic torsional deformities (left subplots, PA01-PA05) and typically developing children (right subplots, TD01-TD05). NSA, FV and TT were quantified from magnetic resonance images using commonly used three-dimensional measurement techniques [36,37]. Simulations for the right leg were performed for each participant.


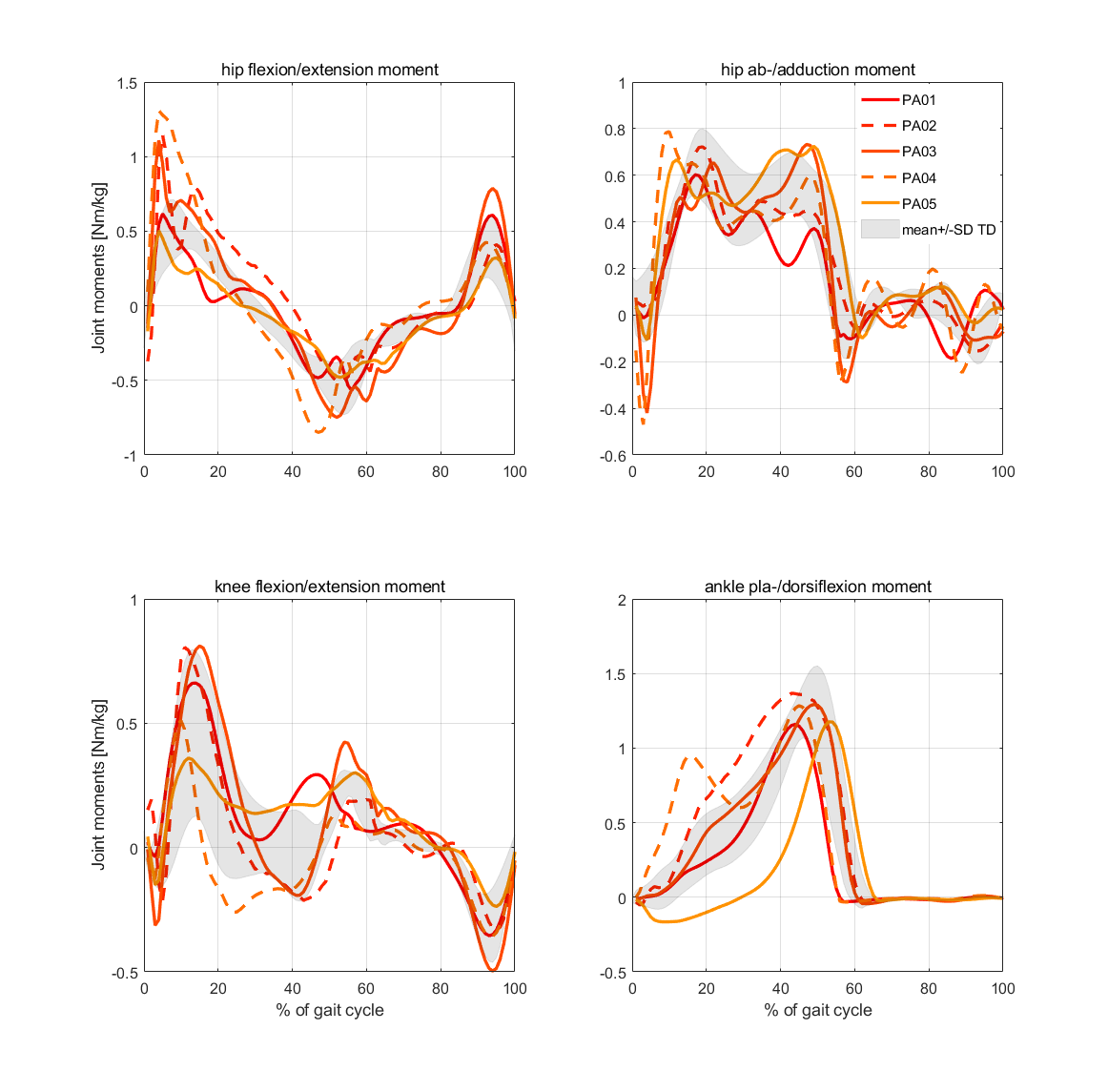


**Figure S2.** Joint moments of the participant with idiopathic torsion deformities.


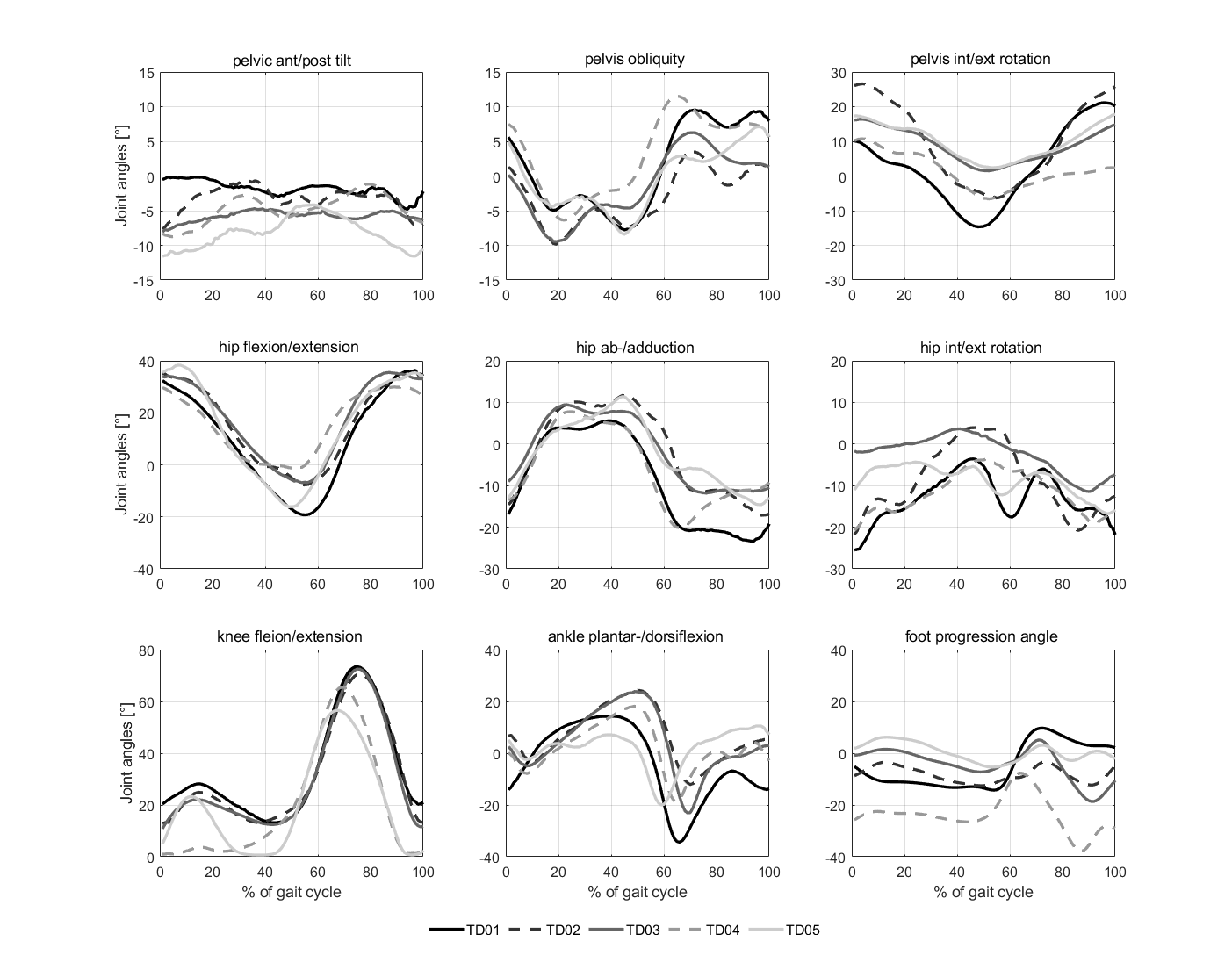


**Figure S3.** Joint kinematics of the TD children.


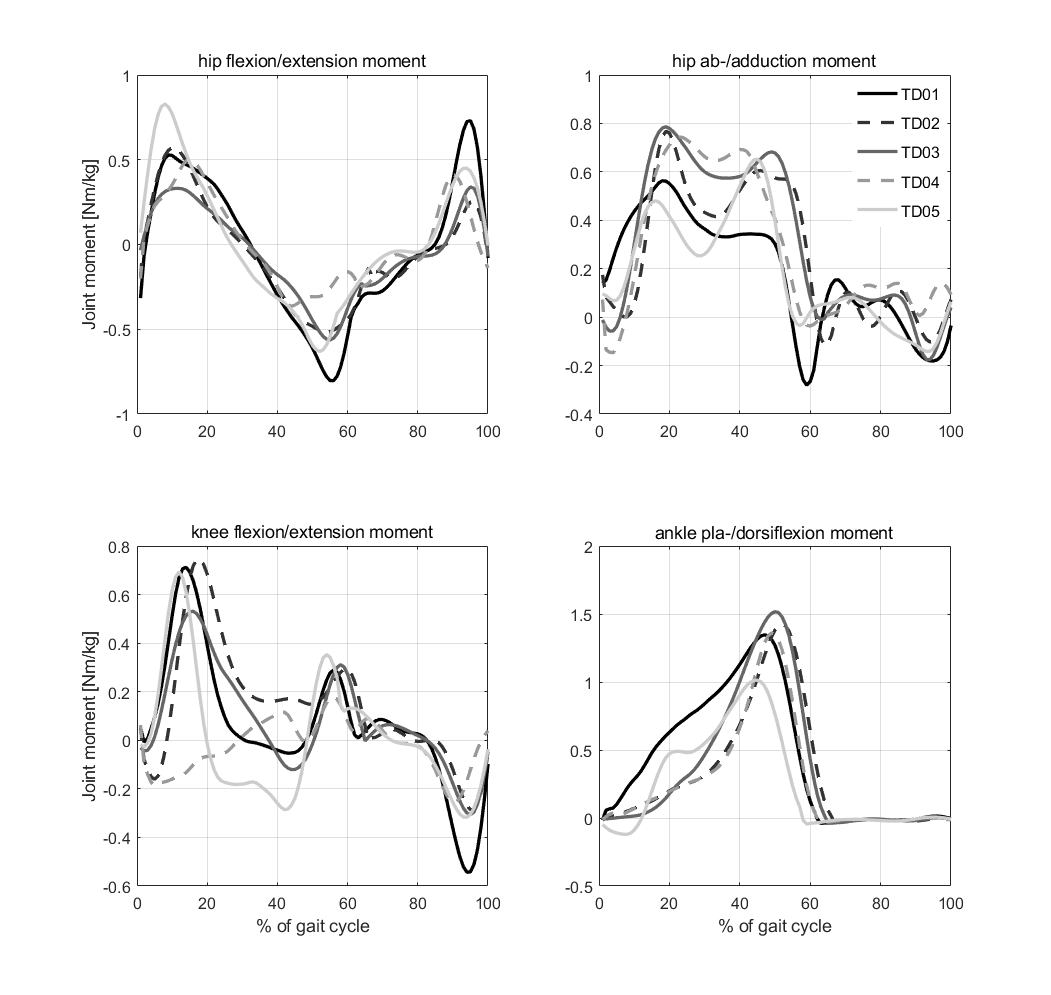


**Figure S4.** Joint moments of the TD children.


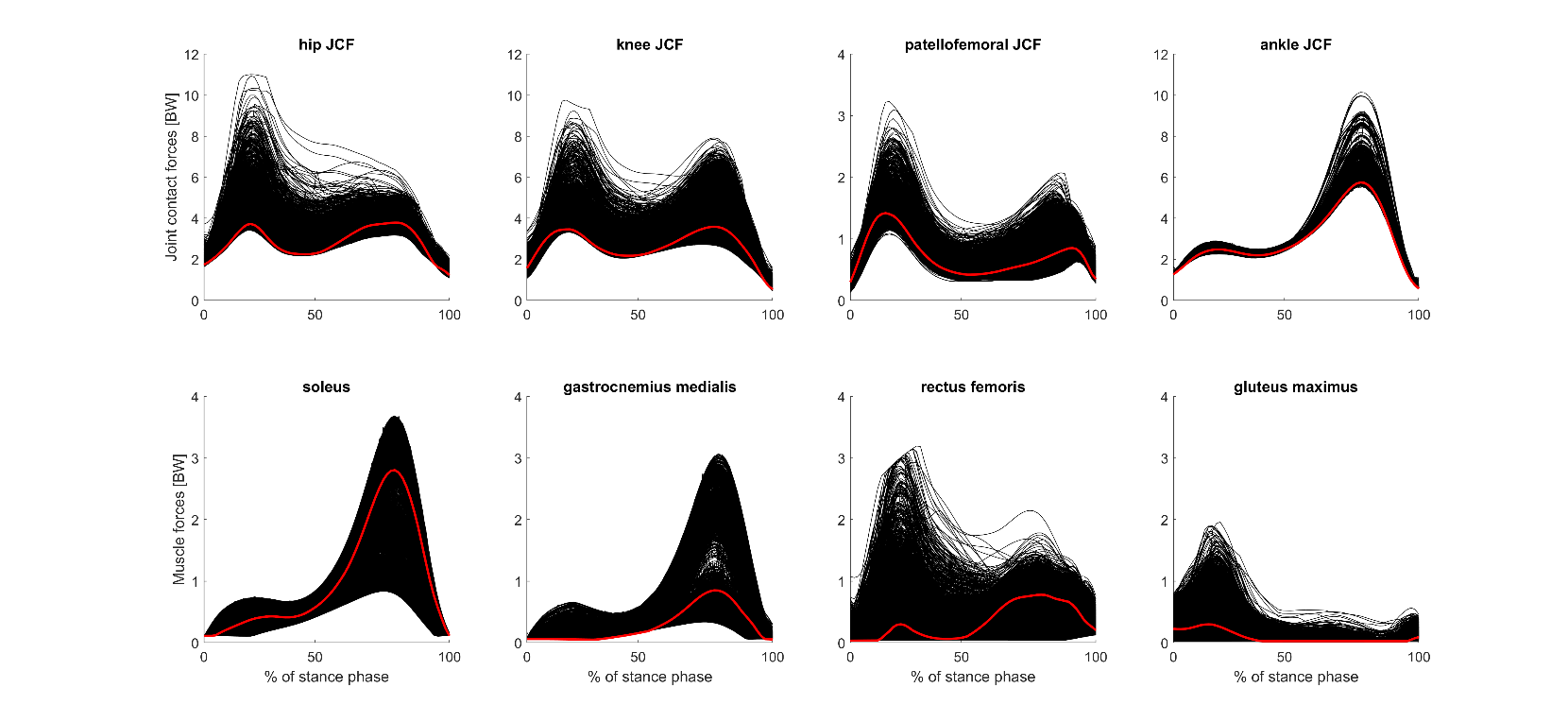


**Figure S5.** Joint contact forces (JCF) and an example of muscle forces obtained from one participant (TD02). Black waveforms are the results from the 10,000 Monte Carlo simulations. Red waveforms are the results from the reference simulation.


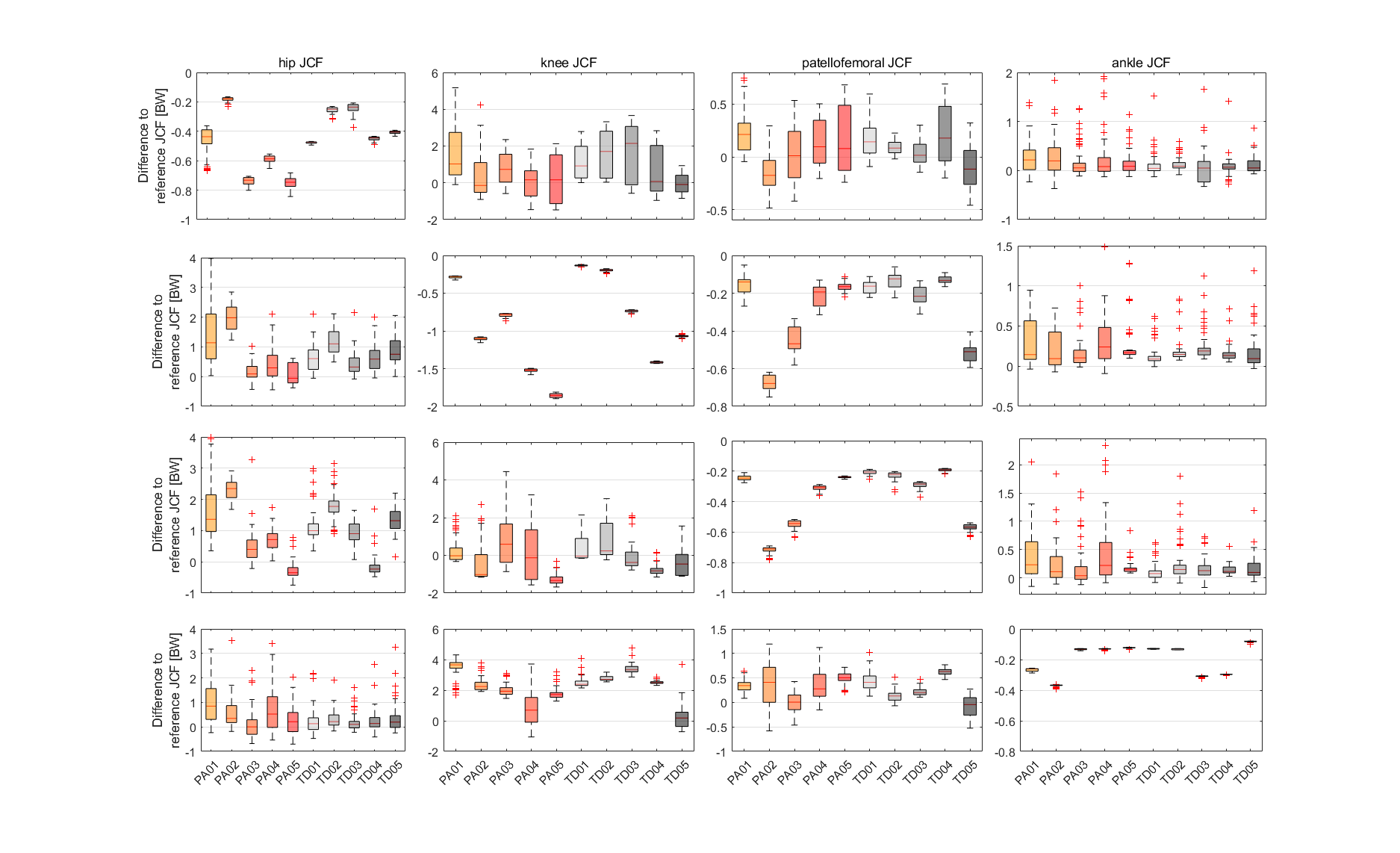


**Figure S6.** Difference to reference joint contact forces (JCF) for simulations with the 50 lowest hip (first row), knee (second row), patellofemoral (third row) and ankle (last row) JCF and the influence on JCF at other joints. Each row of subplots is based on the same simulations.


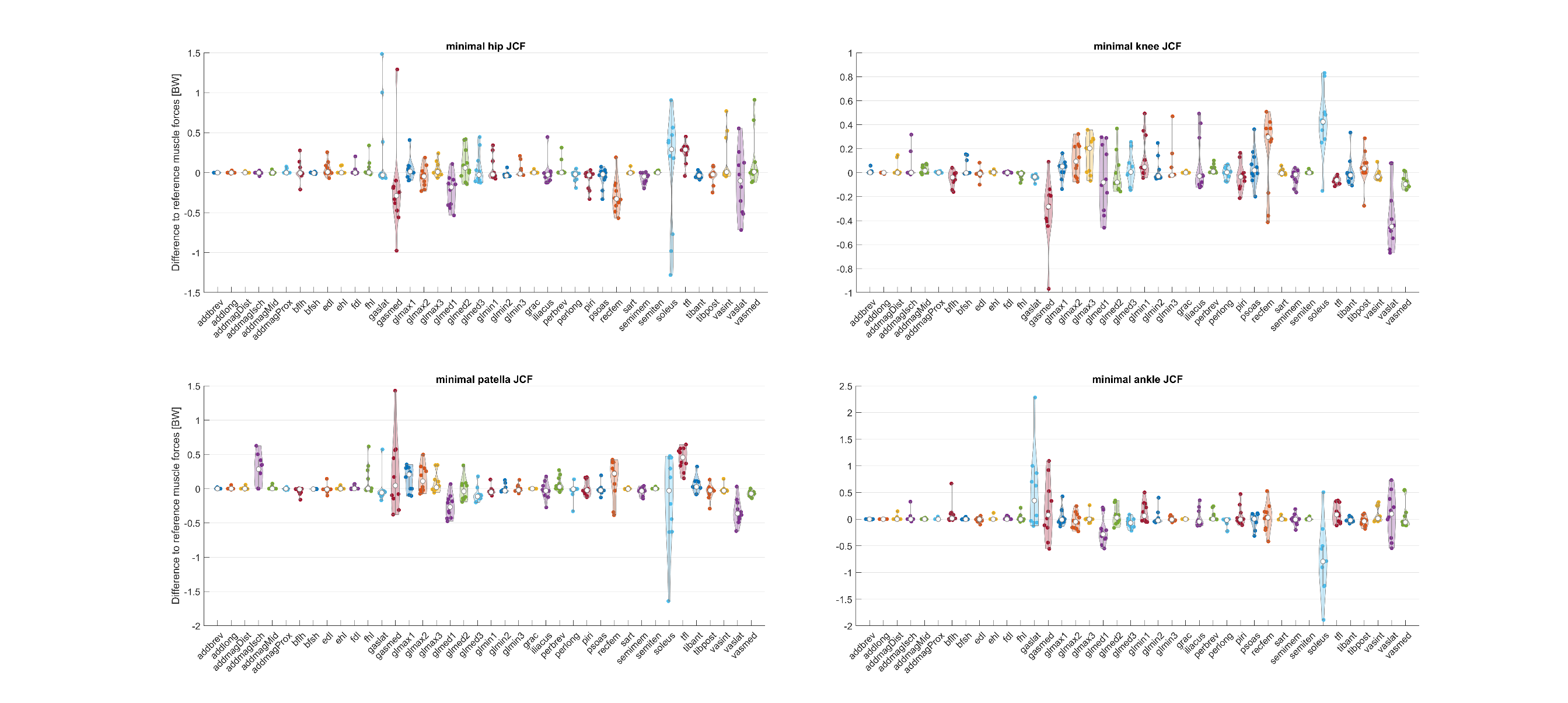


**Figure S7.** Difference in RMS muscle forces to reference muscle forces for simulations with the lowest joint contact forces.


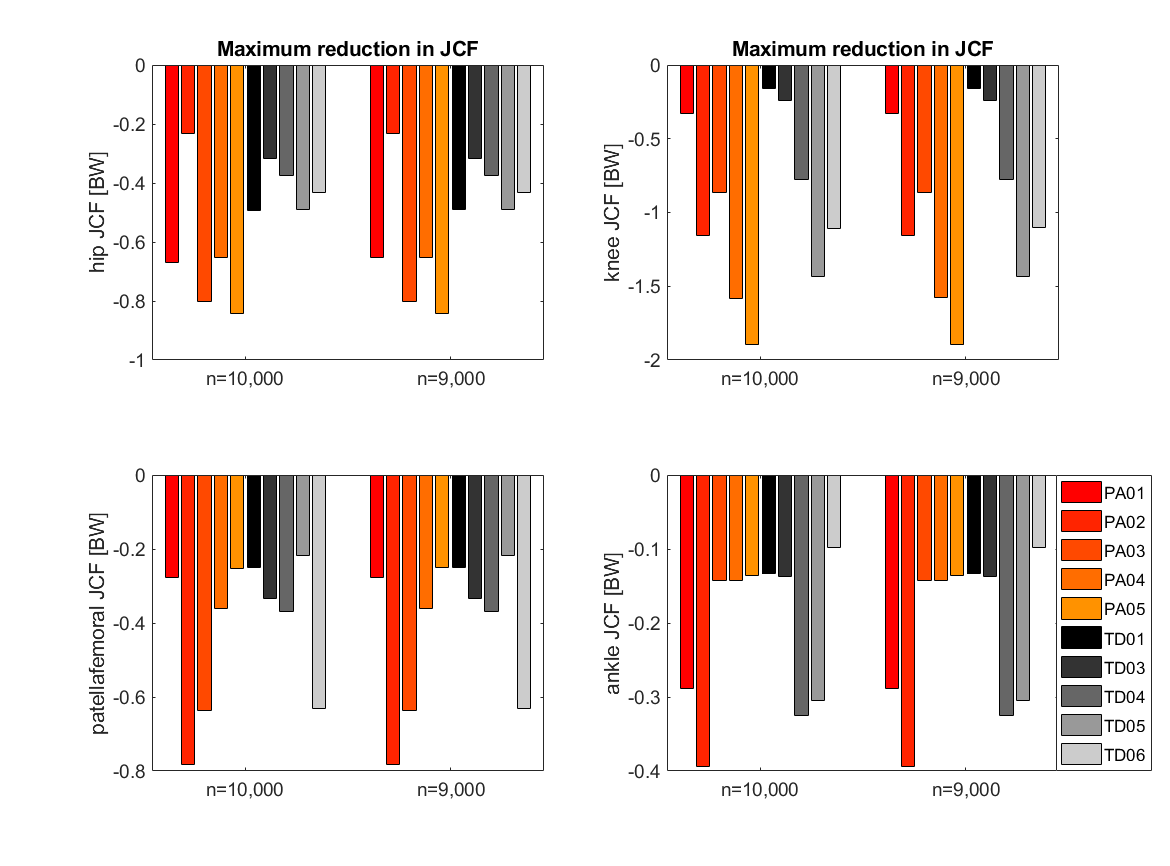


**Figure S8.** Maximum reduction in joint contact forces (JCF) obtained from the Monte Carlo simulations with n=10,000 and n=9,000 simulations. Mean (standard deviation) difference between analysis with n=10,000 and n=9,000 simulations were 0.002 (0.0052) body weight (BW), 0.0007 (0.0015) BW, 0.0001 (0.0004) BW, and 0.00004 (0.0001) BW for hip, knee, patellofemoral and ankle JCF, respectively.

**
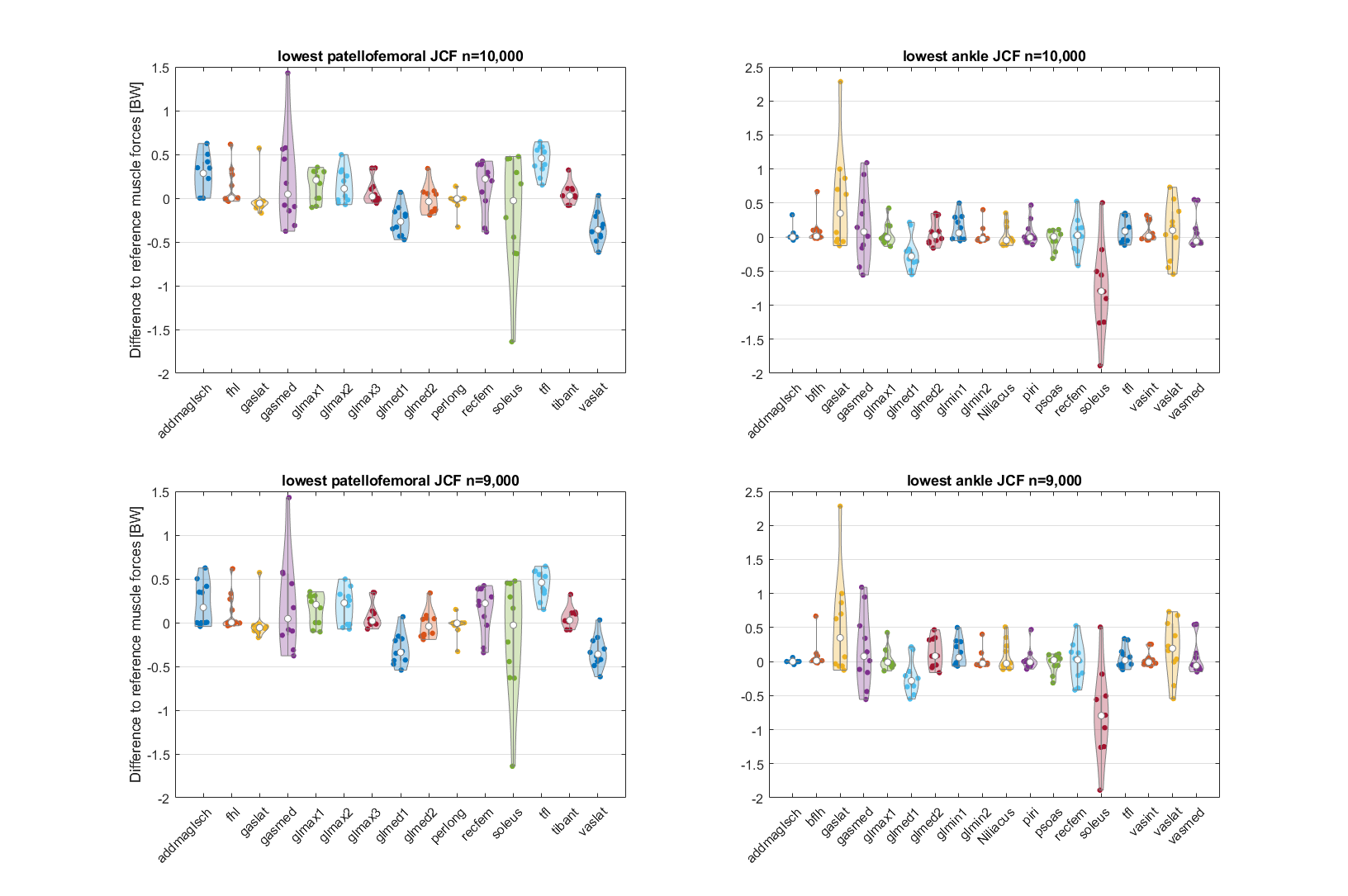
**

**Figure S9.** Difference to reference root-mean-square (RMS) muscle forces for simulations with the lowest patellofemoral and ankle joint contact forces (JCF) obtained from the Monte Carlo analysis with 10,000 (top row) and 9,000 simulations (bottom row). For the majority of participants barely any difference was observed between the analyses based on 10,000 and 9,000 simulations. Each dot in the violin plot represents the result from one participant. FHL = flexor hallucis longus; TFL = tensor fasciae latae.

**
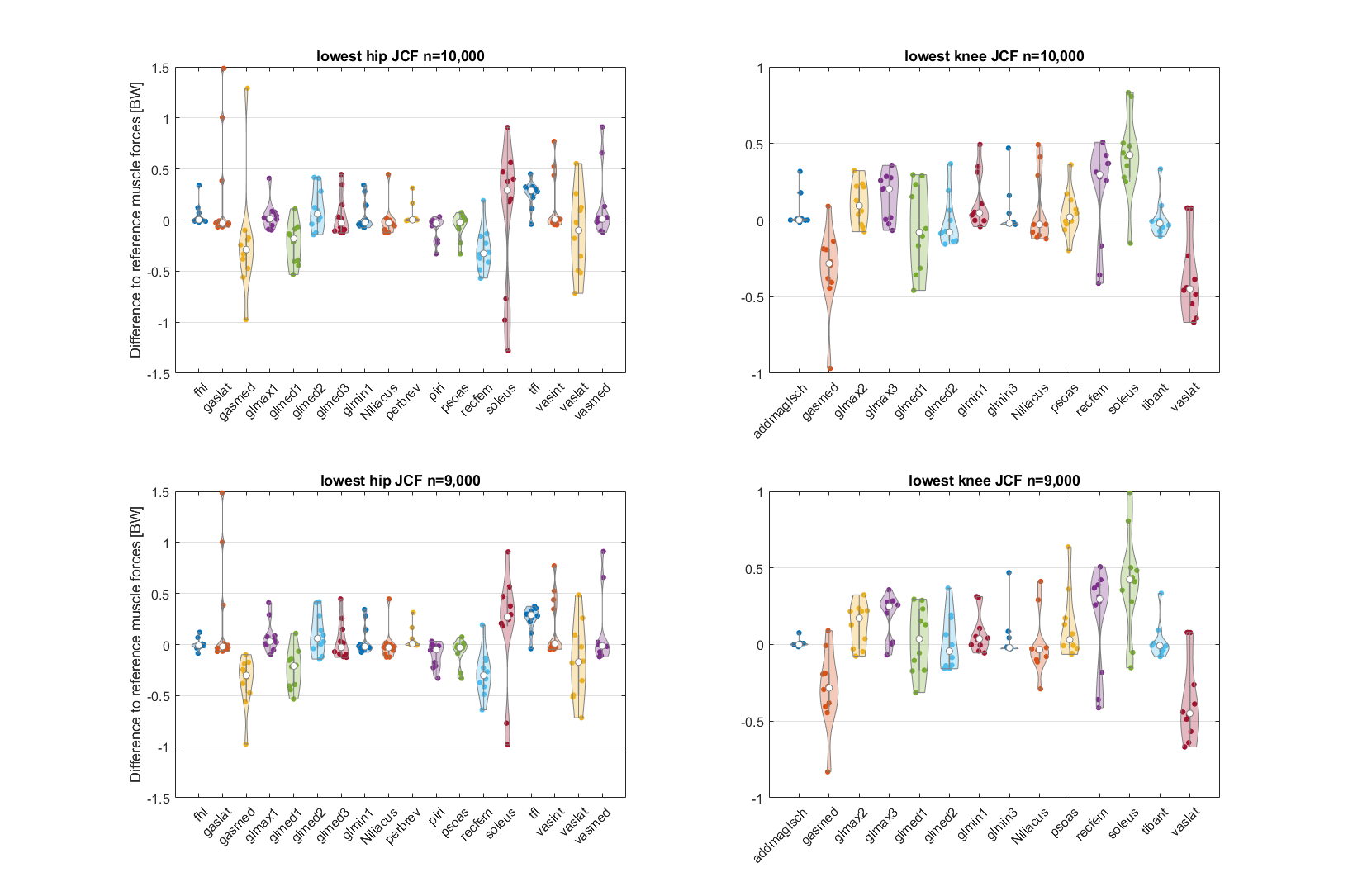
**

**Figure S10.** Difference to reference root-mean-square (RMS) muscle forces for simulations with the lowest hip and knee joint contact forces (JCF) obtained from the Monte Carlo analysis with 10,000 (top row) and 9,000 simulations (bottom row). For the majority of participants barely any difference was observed between the analyses based on 10,000 and 9,000 simulations. Each dot in the violin plot represents the result from one participant. FHL = flexor hallucis longus; TFL = tensor fasciae latae.


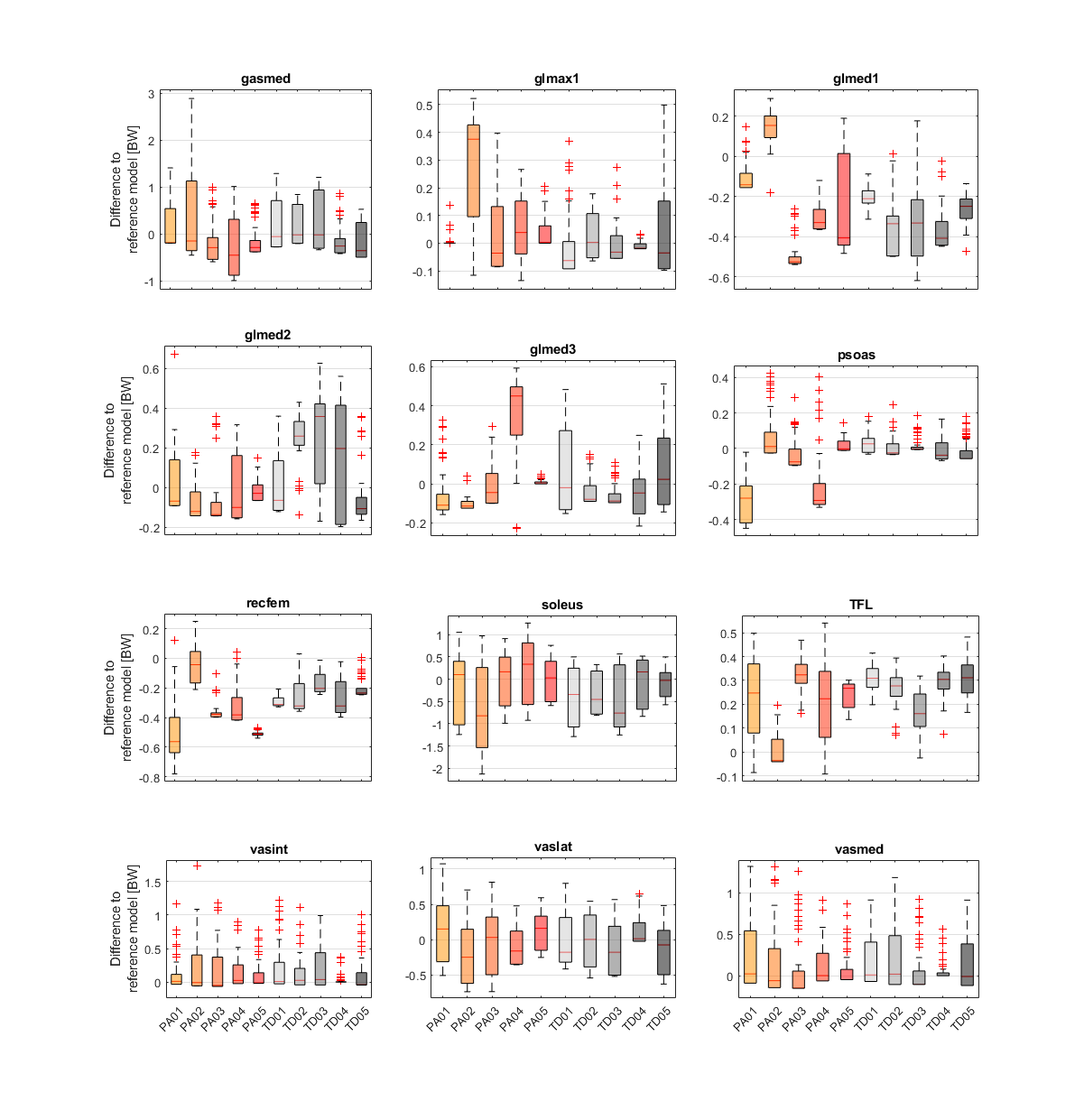


**Figure S11.** Selection of muscles. Difference to reference muscle forces for n=50 simulations with the lowest hip joint contact forces.


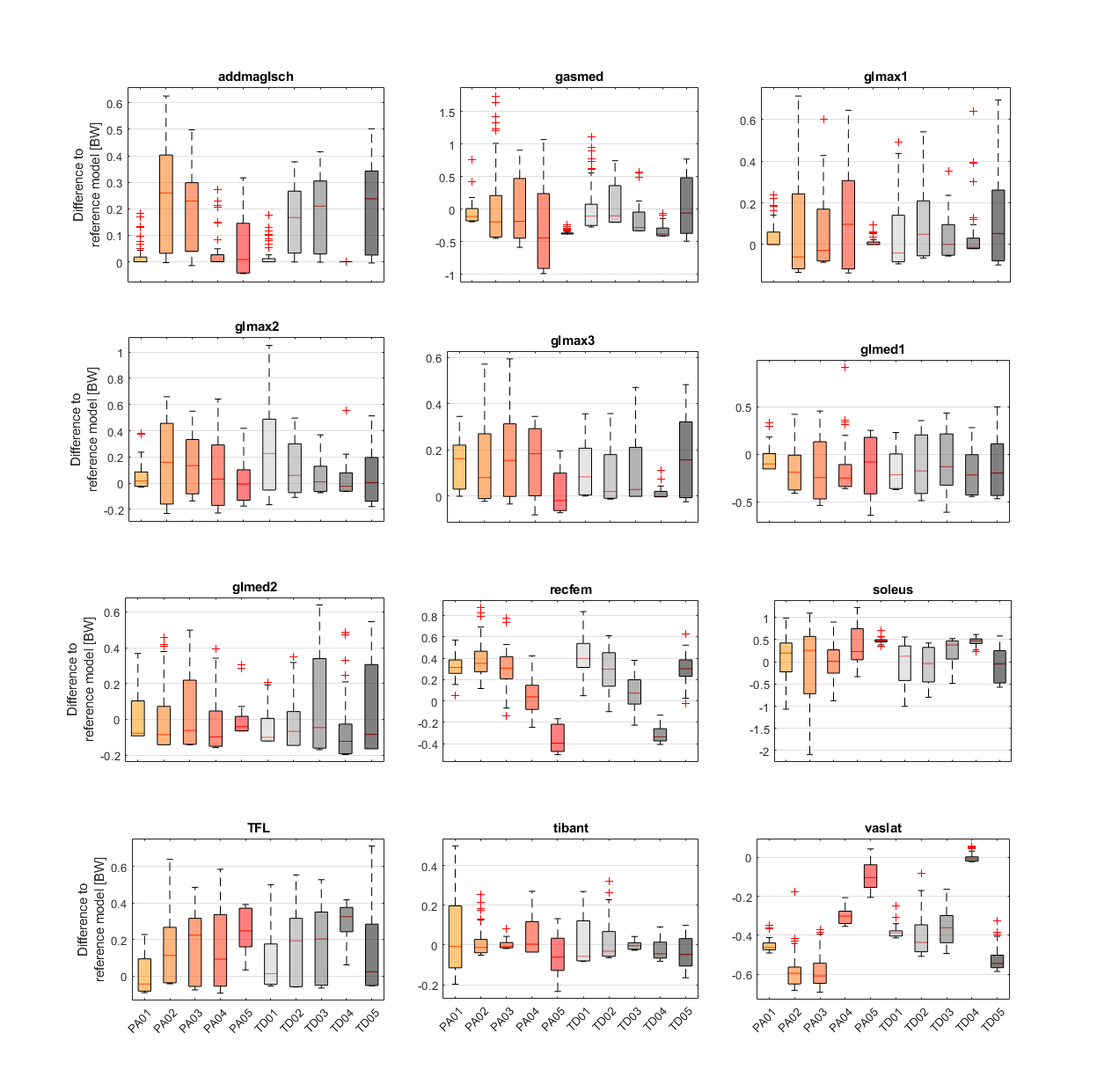


**Figure S12.** Selection of muscles. Difference to reference muscle forces for n=50 simulations with the lowest patellofemoral joint contact forces.


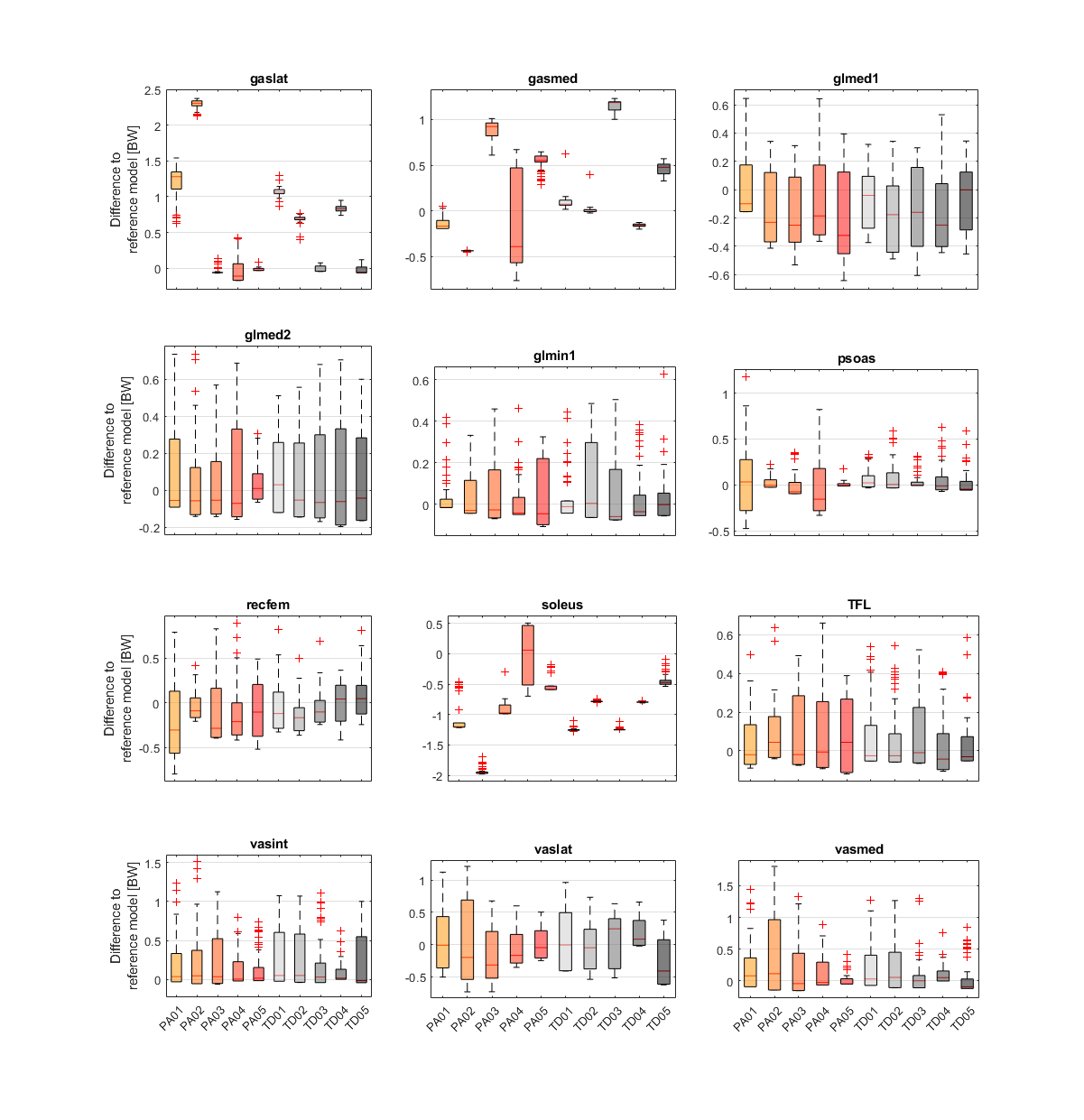


**Figure S13.** Selection of muscles. Difference to reference muscle forces for n=50 simulations with the lowest ankle joint contact forces.


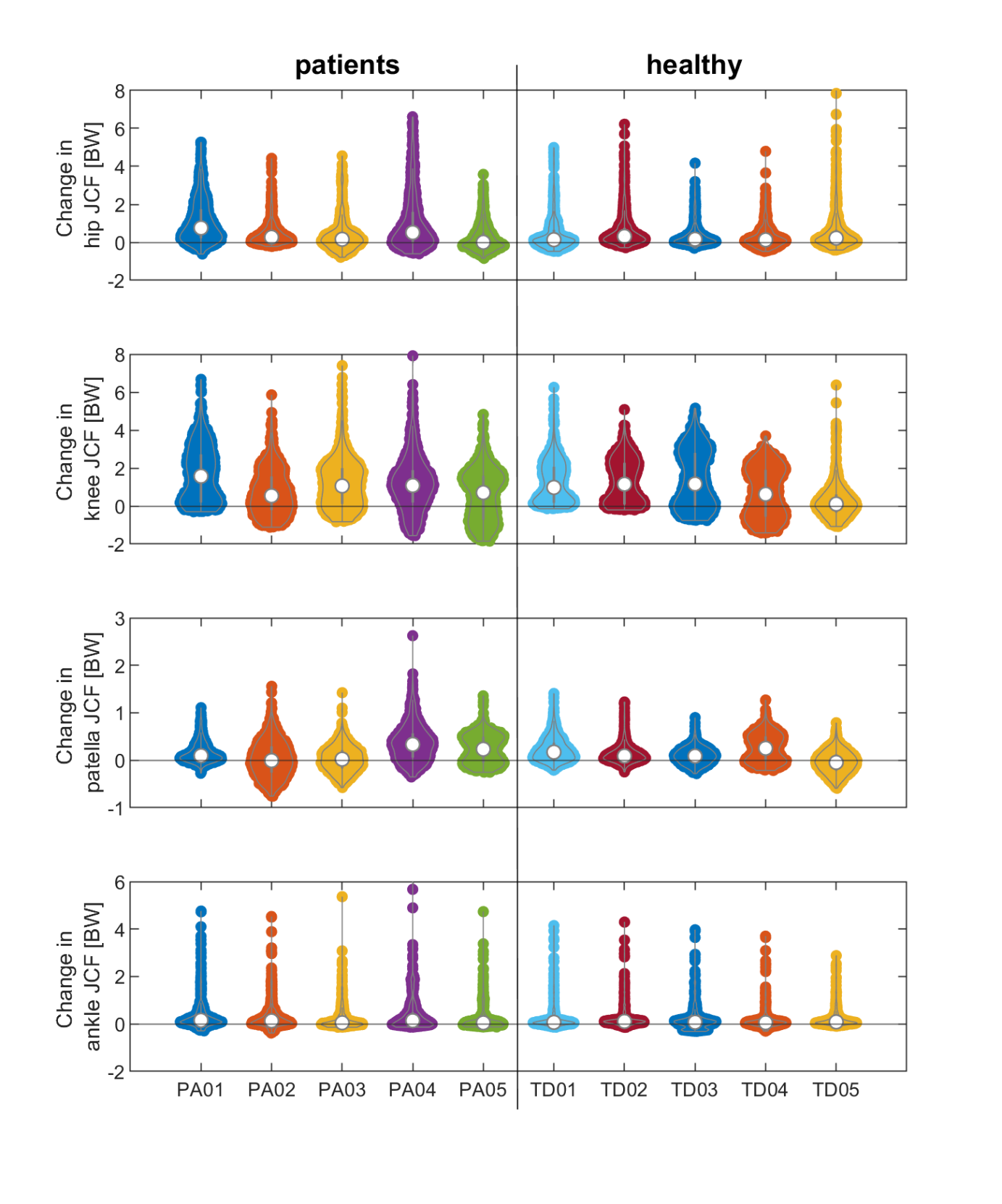


**Figure S14.** Change in joint contact forces (JCF) compared to the reference simulations.


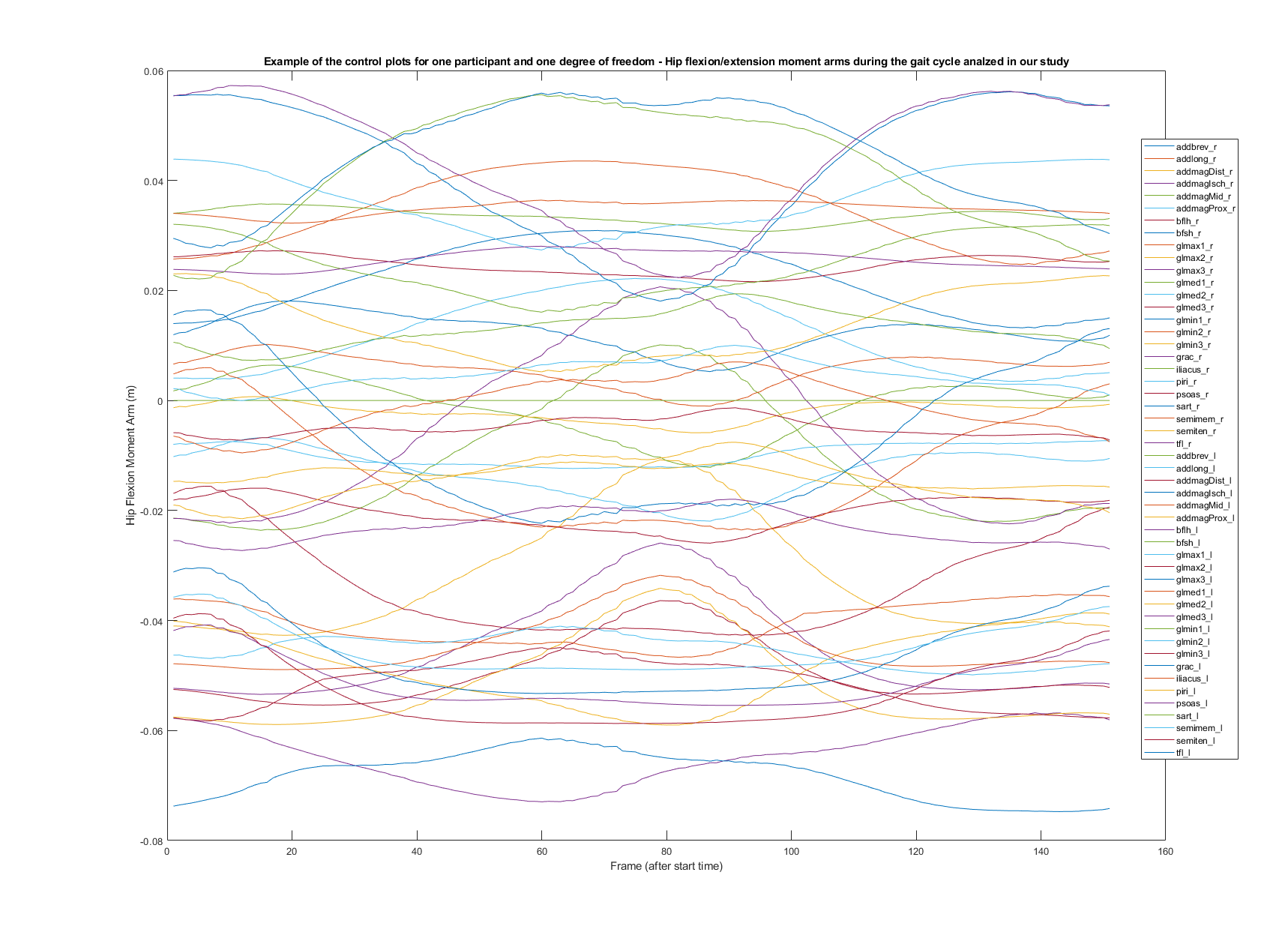


**Figure S15.** Example of control plots used to verify that the muscle-tendon kinematics is reasonable, i.e. does not lead to discontinuities during the walking pattern of the participants analyzed in our study. We used a Matlab script to automatically check if the moment arms and muscle length lead to discontinuities. The script (checkMuscleMomentArms.m) is available on the github folder related to our manuscript (<https://github.com/HansUniVie/MuscleCoordinationRetrainingVie/MuscleCoordinationRetraining>) to enable peers to reproduce our findings and evaluate the quality of the musculoskeletal kinematics of their own models and simulations.


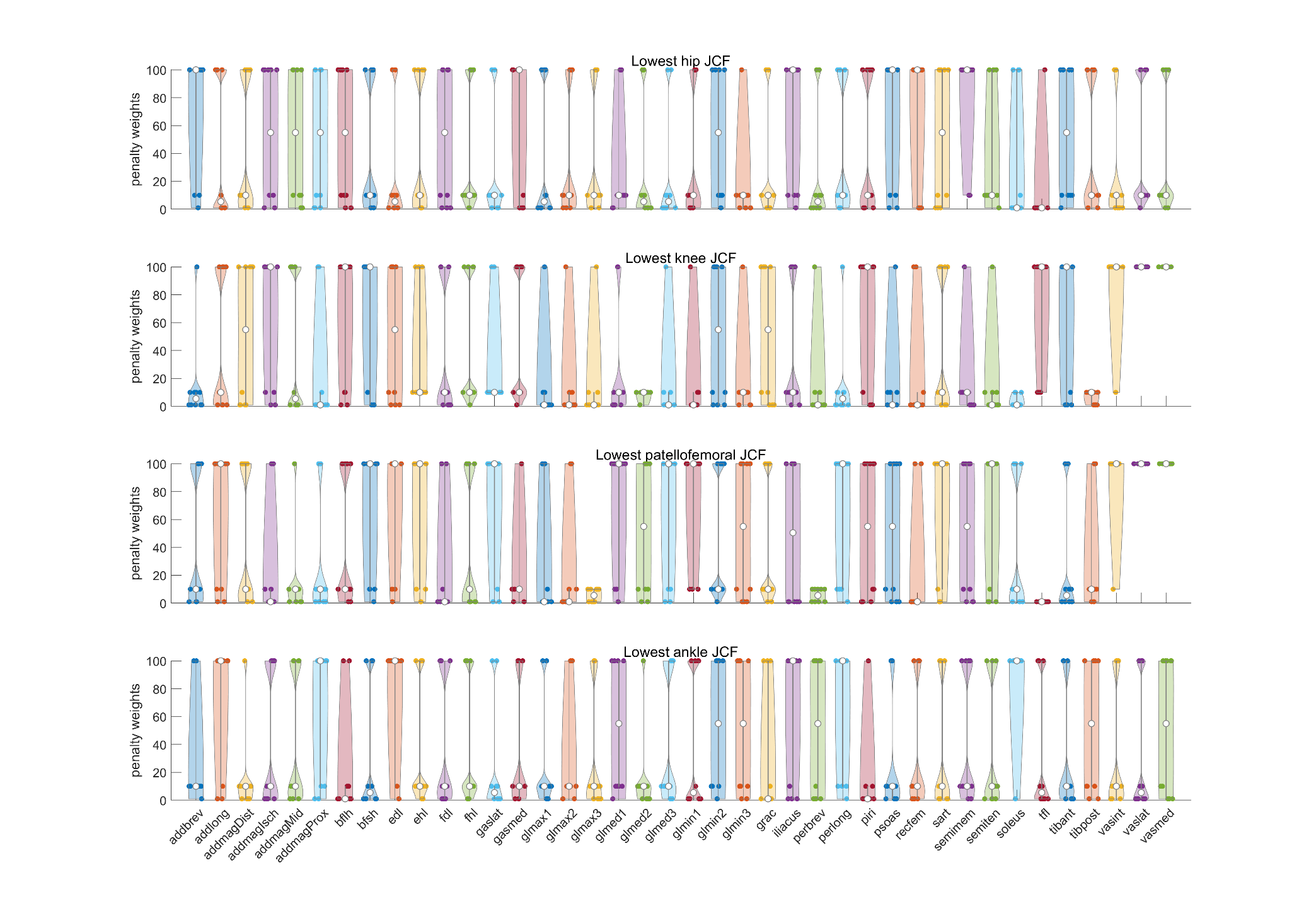


**Figure S16.** Penalty weights for simulations with the lowest joint contact forces (JCF). Each dot in the violin plot represents the weightings from one participant.


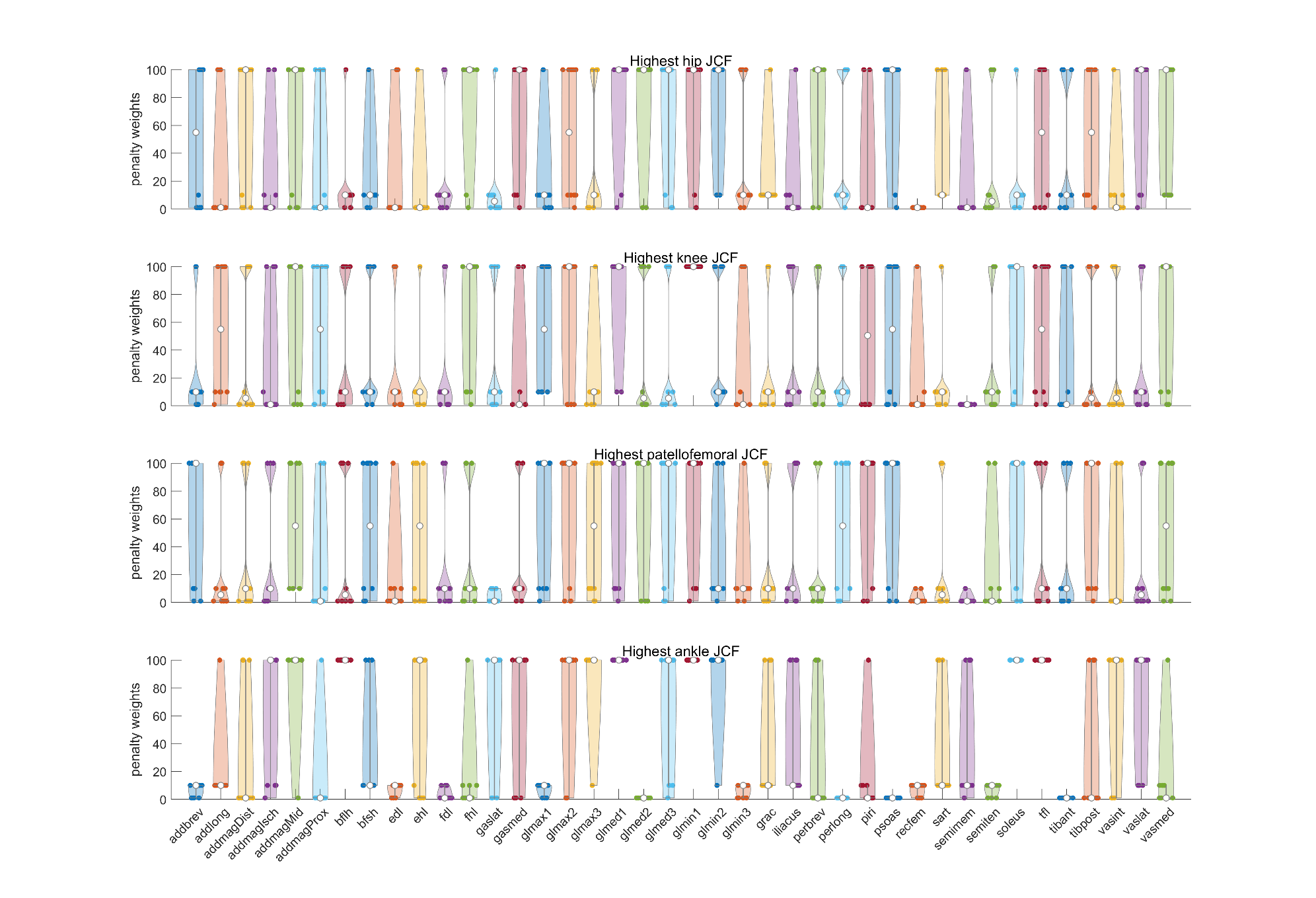


**Figure S17.** Penalty weights for simulations with the highest joint contact forces (JCF). Each dot in the violin plot represents the weightings from one participant.
